# Neuronal activity induces glucosylceramide that is secreted via exosomes for lysosomal degradation in glia

**DOI:** 10.1126/sciadv.abn3326

**Published:** 2022-07-13

**Authors:** Liping Wang, Guang Lin, Zhongyuan Zuo, Yarong Li, Seul Kee Byeon, Akhilesh Pandey, Hugo J. Bellen

**Affiliations:** ^1^ Program in Developmental Biology, Baylor College of Medicine, Houston, TX 77030, USA.; ^2^ Jan and Dan Duncan Neurological Research Institute, Texas Children’s Hospital, Houston, TX 77030, USA.; ^3^ Department of Molecular and Human Genetics, Baylor College of Medicine, Houston, TX 77030, USA.; ^4^ Department of Neuroscience, Baylor College of Medicine, Houston, TX 77030, USA.; ^5^ Department of Laboratory Medicine and Pathology, Mayo Clinic, Rochester, MN 55905, USA.; ^6^ Center for Individualized Medicine, Mayo Clinic, Rochester, MN 55905, USA; ^7^ Manipal Academy of Higher Education, Manipal, Karnataka 576 104, India.

## Abstract

Recessive variants in *GBA1* cause Gaucher disease, a prevalent form of lysosome storage disease. *GBA1* encodes a lysosomal enzyme that hydrolyzes glucosylceramide (GlcCer) into glucose and ceramide. Its loss causes lysosomal dysfunction and increased levels of GlcCer. We generated a null allele of the *Drosophila* ortholog *Gba1b* by inserting the *Gal4* using CRISPR-Cas9. Here, we show that *Gba1b* is expressed in glia but not in neurons. Glial-specific knockdown recapitulates the defects found in *Gba1b* mutants, and these can be rescued by glial expression of human *GBA1*. We show that GlcCer is synthesized upon neuronal activity, and it is transported from neurons to glia through exosomes. Furthermore, we found that glial TGF-β/BMP induces the transfer of GlcCer from neurons to glia and that the White protein, an ABCG transporter, promotes GlcCer trafficking to glial lysosomes for degradation.

## Teaser

Glucosylceramide is synthesized upon neuronal activity and transported to glia via exosomes for degradation by Gba1b and White.

## INTRODUCTION

The cell membrane of eukaryotic cells contains three major classes of lipids: phospholipids (mostly phosphatidylethanolamine and phosphatidylcholines), cholesterol, and sphingolipids. Sphingolipids serve as structural membrane components of cells, and many sphingolipids and their metabolites function in modulating diverse cellular processes (*1*). Although sphingolipids correspond to a relatively small fraction of lipid species, they are far more diverse than phospholipids (*2*).

The most elementary sphingolipid backbone, ceramide, is synthesized in the endoplasmic reticulum from acyl-CoA and serine. After synthesis, it is transported to the Golgi apparatus (Golgi) by the ceramide transfer protein (CERT) for further modification into glycosphingolipids and sphingomyelins (*3*, *4*), which are then transported from the Golgi to the plasma membrane. These lipids modulate the stiffness of the plasma membrane (*3*). A severe reduction in ceramides, as observed upon the loss of CERT in *Drosophila*, increases the fluidity of plasma membranes and the susceptibility to reactive oxygen species (*5*) and severely reduces life span (*6*). In contrast, excessive levels of ceramides form gel-like membrane domains due to aggregation with other phospholipids (*7*, *8*). This leads to reduced membrane fluidity and impaired membrane curvature (*9*). In *Drosophila*, ceramide accumulation in mitochondria negatively affects the mitochondrial function in a Parkinson’s disease (PD) model (*10*). Also, increased levels of ceramides impair membrane endocytosis in aged flies and lead to a reduction of life span (*11*). Moreover, a disequilibrium between ceramide and sphingomyelin due to disruption of ceramidases affects vesicle fusion, trafficking, and synaptic transmission at presynaptic terminals (*12*). Hence, either increased or decreased levels of ceramides affect neuronal homeostasis by regulating membrane fluidity.

Compared to the diversity of ceramides, glycosphingolipids are even more complex. There are hundreds of different forms of glycosphingolipids that are most prevalent in lipid rafts where they play important roles in cell signaling. Glucosylceramide (GlcCer) and galactosylceramide are the two major categories of glycosphingolipids (*2*, *4*). The precise biological function of the vast majority of these lipids is ill-defined, yet knockout of many glycosphingolipid synthases in mice has revealed important functions for the different glycosphingolipids (*13*). Moreover, dysregulation of several glycosphingolipids is found in patients with severe childhood-onset diseases (*14*, *15*). Autosomal recessive variants in *Glucosylceramides Beta* (*GBA1*) cause Gaucher disease (GD), the most prevalent lysosomal storage disease. *GBA1* encodes a lysosomal hydrolase, β-glucosylceramidase, that hydrolyzes GlcCer into glucose and ceramide. Loss of *GBA1* can manifest in infants (type II GD), leading to severe neurological symptoms and death before the age of two (*16*, *17*). Many GD-associated *GBA1* variants have been linked to PD (*14*, *15*, *18*–*20*). These findings indicate a close relationship between glycosphingolipid metabolism and rare as well as common neurological disorders.

Studies in flies (*21*–*23*), fish (*24*), and mice (*25*–*27*) have shown that loss of *GBA1* orthologs leads to GlcCer accumulation, lysosomal dysfunction, ubiquitinated protein aggregation, mitochondrial dysfunction, loss of dopaminergic neurons, progressive locomotor defects, and a shorter life span. However, despite the association between elevated GlcCer levels and neurodegeneration, many key questions remain unanswered. These include the source and dynamics of GlcCer production and degradation at the cellular level. The precise role of GlcCer in GD pathophysiology is also not fully understood.

There are two orthologs of human *GBA1* in *Drosophila*: *Gba1a* and *Gba1b*. Expression data from modENCODE (*28*) and FlyAtlas (*29*) indicate that *Gba1a* is predominantly expressed in the midgut, whereas *Gba1b* is expressed in the brain. In this study, we focus on *Gba1b*. We show that *Gba1b* is expressed and required in glia, but not in neurons, and that glial-specific expression of human *GBA1* rescues systemic loss of *Gba1b* in flies. We also demonstrate that neuronal activity triggers the synthesis of GlcCer in neurons, which is then secreted from neurons via exosomes. GlcCer released from neurons is then taken up by glia for lysosomal degradation. Loss of the *white* gene, which encodes an ABCG transporter abundantly expressed in pigment glia (*30*, *31*), severely affects the glial lysosomal degradation of GlcCer. Last, we present compelling evidence that the mechanism of transport of GlcCer from neurons to glia via exosomes is conserved in vertebrate neuronal cells and depends on transforming growth factor–β (TGF-β)/bone morphogenetic protein (BMP) signals produced in glia.

## RESULTS

### *Gba1b* is expressed in glial cells but not in neurons

**Fig. 1.**
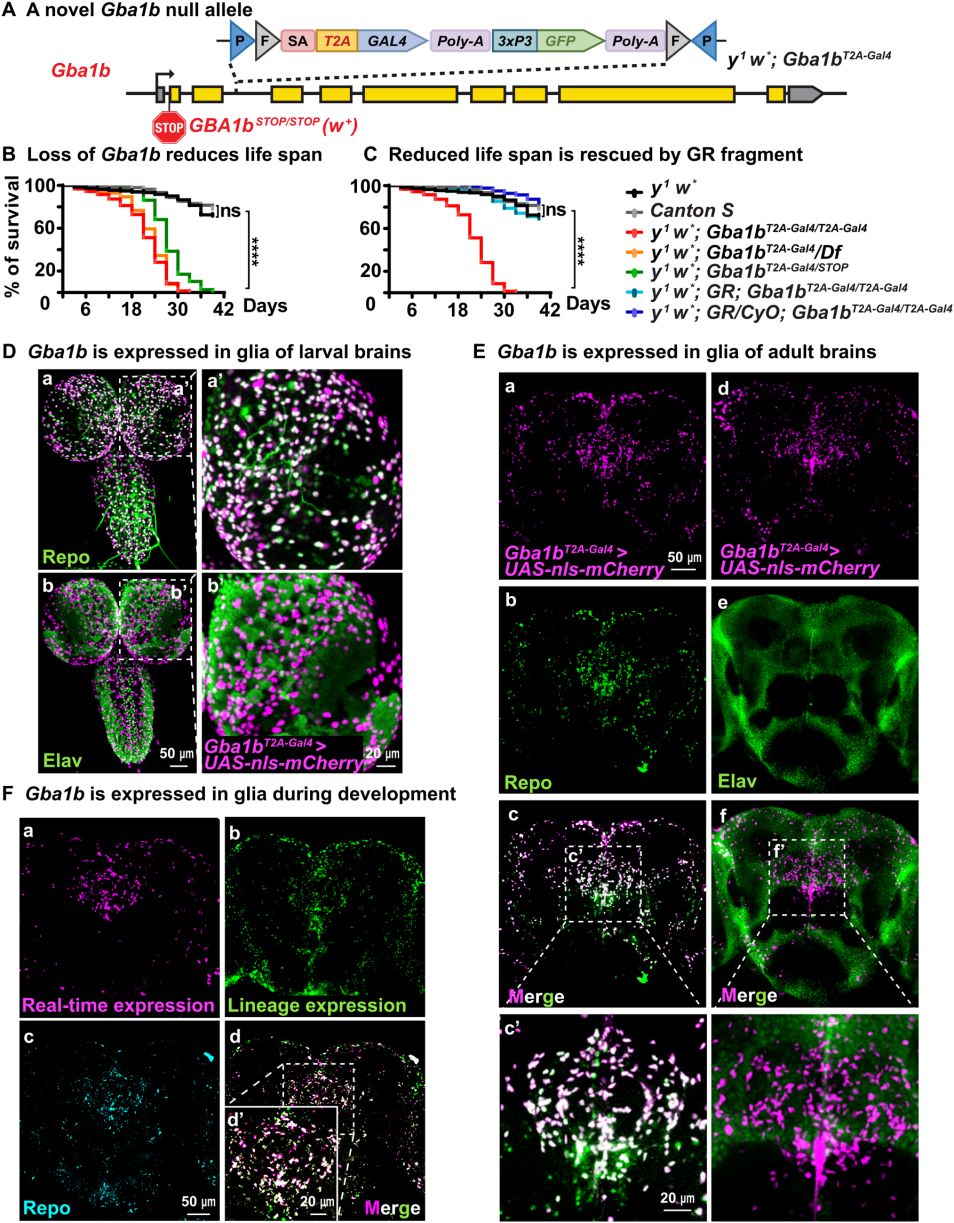
*Gba1b* is expressed in glia. (**A**) y^1^ w*; Gba1b^T2A-Gal/T2A-Gal4^: A construct containing *attp-FRT-SA-T2A-GAL4-PolyA-3xP3GFP-FRT-attp* was inserted in *Gba1b* using CRIMIC technology (*32*). This insertion creates a null allele as shown in fig. S1. It also leads to the production of GAL4 protein in the same temporal and spatial expression pattern as *Gba1b*. (**B** and **C**) Reduced life span of flies that lack *Gba1b*: These include (i) y^1^ w*; Gba1b^T2A-Gal4/T2A-Gal4^, (ii) y^1^ w*; Gba1b^T2A-Gal4^/Df, and (iii) y^1^ w*; Gba1b^T2A-Gal/STOP^ (this is a red-eyed fly due to the presence of the *Gba1b ^STOP^* allele). The *Gba1b^STOP/STOP^* allele contains an early stop codon and an insertion of the *mini-white^+^* gene (*22*). Reduced life span can be fully rescued by one or two copies of a genomic fragment (GR) that contains the *Gba1b* gene and the *white^+^* gene (*N* > 200). ****P < 0.0001. (**D** and **E**) *Gba1b* is expressed in glia in larval and adult brains. The GAL4 protein produced by the y^1^ w*; Gba1b^T2A-Gal4^ insertion is used to drive the nls::mCherry protein (magenta). On the basis of costaining with Repo (green) present in the nuclei of glia, *Gba1b* is only expressed in glia. Moreover, there is no obvious colocalization between Elav (green), which marks neuronal nuclei, and nls::mCherry, which reports *Gba1b* expression, indicating that *Gba1b* is not expressed in neurons (*N* = 3). (**F**) Magenta and green signals represent real-time and lineage expression of *Gba1b*, respectively (see fig. S1, B and C). Again, *Gba1b* is only expressed in glia. Repo (canyon) labels the glial nuclei (*N* = 3).

To determine the expression pattern of *Gba1b* in the central nervous system (CNS), we used the CRIMIC technology (*32*), as there is no antibody available for *Drosophila Gba1b*. We inserted a CRIMIC cassette containing *SA-T2A-GAL4-PolyA* into the third intron of *Gba1b* (Fig. 1A). This artificial exon drives the expression of GAL4 protein under the control of endogenous *Gba1b* regulatory elements while arresting transcription because of the presence of the *Poly-A* sequence (*33*). Reverse transcription polymerase chain reaction results show that this y^1^ w*; Gba1b^T2A-Gal4^ allele is a null allele (fig. S1A). Other alleles include *w^1118^; dGBA1b*^−/−^ (abbreviated as *Gba1b ^STOP^*), a severe loss-of-function allele that carries the *mini-white^+^* gene (*22*), and *w^1118^; Df(3R)BSC490/TM6C, Sb^1^ cu^1^* (*Df* for short), which uncovers *Gba1b* and several other genes (*34*). Flies that lack *Gba1b* (y^1^ w*; Gba1b^T2A-Gal/T2A-Gal4^, y^1^ w*; Gba1b^T2A-Gal4^/*Df*, and y^1^ w*; Gba1b^*T2A-Gal4*/*STOP*^) show a severely reduced life span when compared with y^1^ w^*^ and *Canton S* flies (Fig. 1B). The reduced life span caused by the loss of *Gba1b* is fully rescued by the introduction of one or two copies of a 20-kb *P{acman; CH322-118C10}* (*35*), a genomic bacterial artificial chromosome (BAC) that contains the *Gba1b* and the *mini-white^+^* gene as a dominant marker (Fig. 1C). The total GlcCer levels are increased 16-fold in y^1^ w*; Gba1b^T2A-Gal/T2A-Gal4^ mutant brains when compared to y^1^ w* controls (fig. S1B), consistent with what has been documented for the *Gba1b ^STOP^* allele (*22*).

To identify the cells that express *Gba1b* in the CNS, we used y^1^ w*; Gba1b^T2A-Gal4^ to drive *UAS-nls (nuclear localization signal)::mCherry* (*32*). The mCherry-positive nuclei represent cells that express *Gba1b*, and costaining with an antibody against Elav (a neuronal nuclear marker) (*36*) or Repo (a glial nuclear marker) (*37*) shows that *Gba1b* is expressed in glia of third instar larval (Fig. 1D) and adult brains (Fig. 1E). To determine whether neurons transiently express *Gba1b* during development, we used G-TRACE (*38*). It reports the historical/lineage expression of a gene (fig. S1C). G-TRACE of y^1^ w*; Gba1b^T2A-Gal4^ costaining with Repo or Elav indicates that *Gba1b* is not expressed in neurons during development (Fig. 1F and fig. S1D). Hence, these data demonstrate that *Gba1b* is expressed in glia in the CNS of *Drosophila*.

**Fig. 2.**
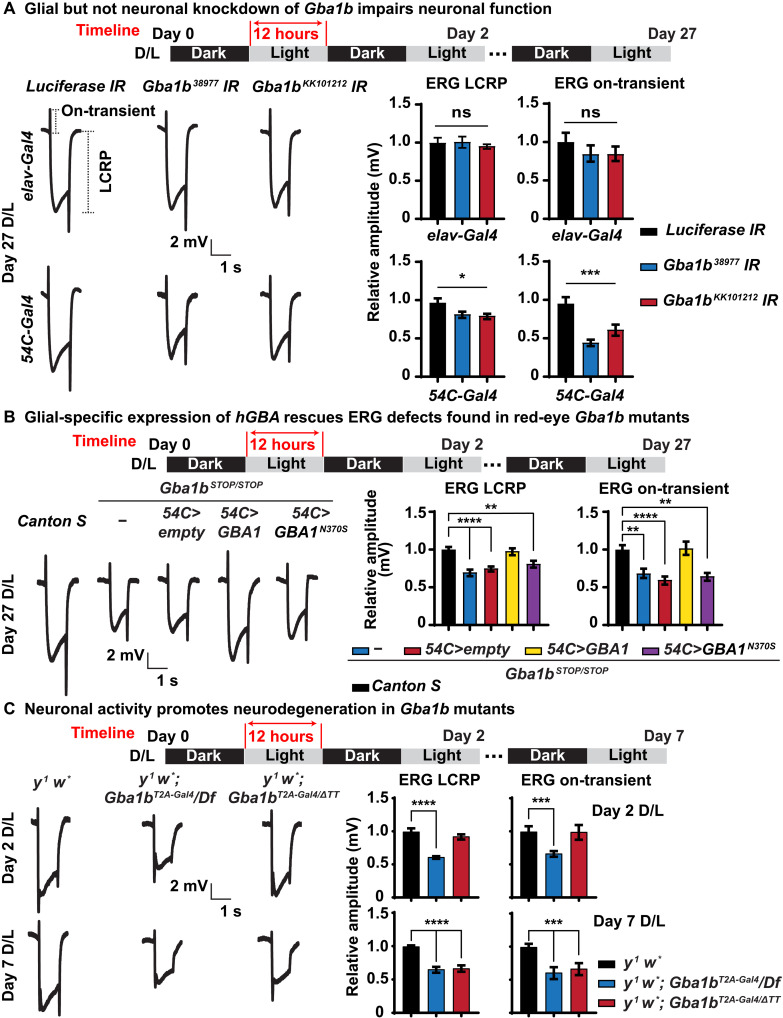
Glial Gba1b is necessary and sufficient to support neuronal function. (**A**) *Gba1b* is required in glia to support neuronal function. ERG recordings of flies of the indicated genotypes after 27 days of D/L cycles. *Gba1b* is specifically knocked down in neurons driven by *elav-Gal4* (top) and in glia driven by *54C-Gal4* (bottom), respectively. Glial knockdown but not neuronal knockdown of *Gba1b* leads to reduction of LCRPs and on-transient. The ERG LCRP and on-transient amplitudes are quantified on the right. Error bars represent SEM (*n* ≥ 6); *P < 0.05 and ***P < 0.001. (**B**) Glial-specific expression of human *GBA1* but not *GBA1^N370S^* rescues ERG phenotypes in *Gba1b* mutants. ERG recordings of flies of the indicated genotypes after 27 days of D/L cycles. *Gba1b^STOP/STOP^* shows reduced ERG LCRP and on-transient, which can be fully rescued by glial expression of *GBA1* driven by *54C-Gal4*. The ERG LCRP and on-transient amplitudes are quantified on the right. Error bars represent SEM (*n* ≥ 6); **P < 0.01 and ****P < 0.0001. Flies that are tested in this experiment are red eyed because of the transgenes. (**C**) Neuronal activity promotes neurodegeneration in *Gba1b* mutants. ERG recordings of flies of the indicated genotypes after 2 and 7 days of D/L cycles. y^1^ w*; Gba1b^Δ*TT*^ is a partial loss-of-function allele with 40% remaining enzymatic activity (*21*). At day 2 (top), loss of *Gba1b* (y^1^ w*; Gba1b^T2A-Gal4^/Df) causes reduced ERG LCRP and on-transient amplitudes, whereas the partial loss-of-function allele (y^1^ w*; Gba1b^*T2A-Gal4/*Δ*TT*^) does not obviously affect the ERGs. At day 7 (bottom), both allelic combinations exhibit reduced LCRP and on-transient amplitudes. The data are quantified on the right. Error bars represent SEM (*n* ≥ 6); ***P < 0.001 and ****P < 0.0001. Flies that are tested in this experiment are phenotypically white.

To explore the function of *GBA1b* in glia, we used the *Drosophila* visual system as our experimental model. The fly eye is composed of ~750 units called ommatidia. Each ommatidium contains eight photoreceptor neurons surrounded by pigment and cone cells, which function as glia (*39*, *40*). We performed knockdowns of *Gba1b* in photoreceptor neurons using the *elav-Gal4* driver (*41*) or in pigment glia using the *54C-Gal4* driver (*39*, *42*). Flies were raised under constant darkness conditions until the third day after eclosion (day 0) and were aged for 27 days (day 27) in a 12-hour dark/12-hour light (D/L) cycle at 2500 lux. To assess neuronal function in adults, we performed electroretinogram (ERG) recordings. ERGs assess the neuronal function of the photoreceptors and synaptic transduction in the visual system (*43*). Upon light exposure, the amplitude of light coincident receptor potential (LCRP) measures the light-dependent phototransduction, whereas the amplitude of the on/off transient is a measure of synaptic transmission between photoreceptors and lamina neurons. As shown in Fig. 2A, glial-specific knockdown of *Gba1b* using RNA interference (RNAi) leads to a reduction of the LCRPs and on-transient. However, no defect is observed upon neuronal knockdown, consistent with the glial-specific expression of *Gba1b*. This indicates that *Gba1b* is required in glial cells to support proper eye function and that its loss affects the phototransduction cascade and synaptic transmission.

To assess whether similar phenotypes were observed in *Gba1b* null mutants, we first recorded ERGs in *Gba1b^STOP/STOP^* mutants and observed similar reduced LCRPs and on-transient (Fig. 2B). Expression of the human *GBA1* reference complementary DNA (cDNA) driven specifically in glia by *54C-Gal4* rescues the ERG defects of *Gba1b^STOP/STOP^* animals (Fig. 2B). However, glial expression of human *GBA1^N370S^*, a missense variant found in patients with GD that retains about 30% of β-glucosylceramidase enzymatic activity (*44*), fails to rescue the ERG defects (Fig. 2B). These data demonstrate that human *GBA1* and fly *Gba1b* functions are evolutionarily conserved.

To determine the phenotype associated with the other *Gba1b* loss-of-function alleles, we performed ERGs on homozygous y^1^ w*; Gba1b^T2A-Gal4^/Gba1b^T2A-Gal4^ and transheterozygous y^1^ w*; Gba1b^T2A-Gal4^/Df. We also tested a partial loss-of-function combination, y^1^ w*; Gba1b^*T2A-Gal4/*Δ*TT*^. The *w^1118^*; *GBA1*^Δ*TT*^ allele is a hypomorphic allele that retains about 40% of β-glucosylceramidase activity in fly brains (*21*). We first raised the mutant flies in constant darkness (D/D). These flies only show a very mild impairment in LCRPs and no defect in on-transient at day 7 (fig. S2A). To determine whether light-induced neuronal activity is required to cause ERG defects, we shifted the flies to the D/L condition. A complete loss of *Gba1b* impairs neuronal activity at day 2 (Fig. 2C), indicating that neuronal activity stimulated by light is required to induce a strong loss-of-function phenotype. However, the partial loss of *Gba1b* does not show ERG defects before day 7 (Fig. 2C). These data show that prolonged light-induced neuronal activity is required to trigger ERG defects when residual enzymatic activity is present. The ERG defects observed in *Gba1b* null flies are fully rescued by glial expression of human *GBA1* reference cDNA but not by *GBA1^N370S^* (fig. S2B). These data show that *Gba1b/GBA1* is required in glia and is sufficient to support normal function.

The above data raise an issue as to why some phenotypes are observed at day 27 whereas other assays reveal phenotype at a much earlier time point at day 2. The assays that reveal phenotypes in young flies (Fig. 2C) were all performed in flies that have white eyes (*white^−^* background), whereas those shown in Fig. 2 (A and B) were performed in red-eye flies (*white^+^* background). Note that the ERG amplitudes of LCRP and on-transient are very similar in y^1^ w^*^ (*white^−^* background) and *Canton S* (*white^+^* background) flies after 7 days of a D/L cycle (fig. S2C), suggesting that the ERG defects shown in Fig. 2C are due to the lack of Gba1b but not loss of pigmentation in the fly eyes. Moreover, as shown in fig. S2C, y^1^ w^*^ flies exhibit reduced ERG LCRP and on-transient amplitude at day 27 compared with *Canton S* flies, indicating that the *white* gene may play a role in this process. We will address this issue below. In summary, all the data presented so far indicate that *Gba1b* is required in glia to support neuronal function.

### Loss of *Gba1b* leads to abnormal glia morphology and progressive neurodegeneration

**Fig. 3.**
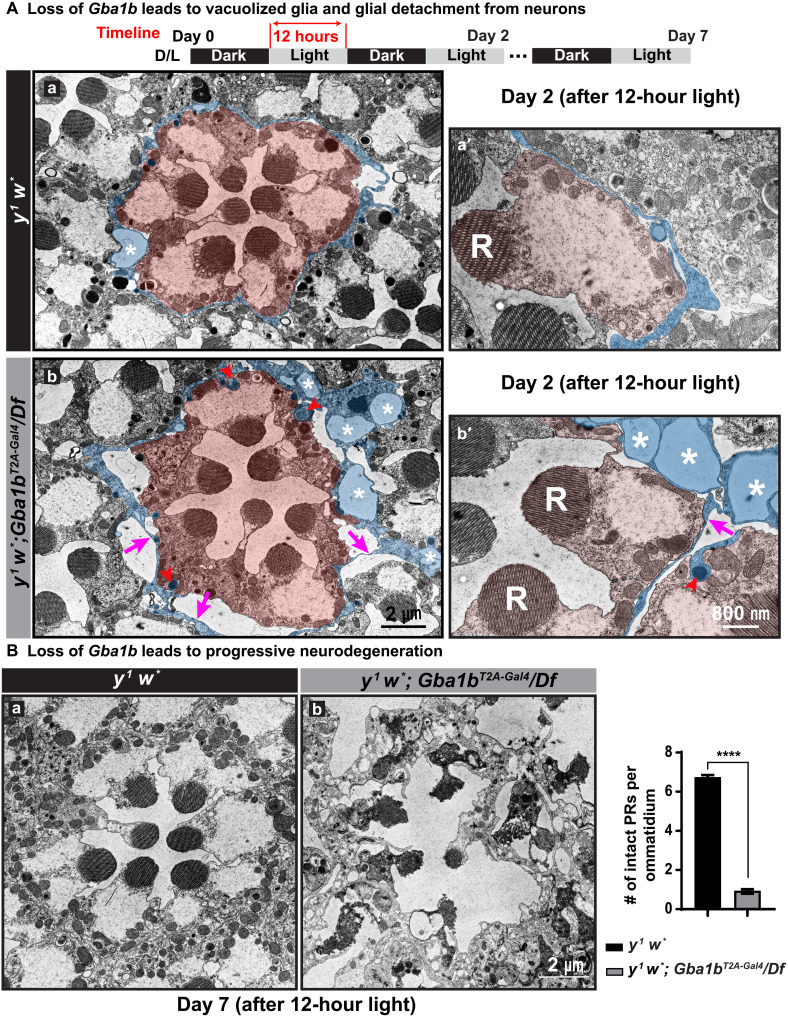
Loss of *Gba1b* impairs glial morphology, which precedes neuronal loss. (**A**) Loss of *Gba1b* leads to vacuolized glia. TEM images of fly retina of the indicated genotypes after 2 days of D/L cycles. Photoreceptor neurons are highlighted in orange, and pigment cells are highlighted in blue (a, a′, b, and b′). R, rhabdomeres. Red arrowheads point to lysosomes in pigment cells. y^1^ w*; Gba1b^T2A-Gal4^/Df null mutants exhibit an increased number of lysosomes in glia when compared to glia of y^1^ w^*^ flies (b and b′). Magenta arrows indicate glial detachment, which is commonly seen in the retina of y^1^ w*; Gba1b^T2A-Gal4^/Df null mutants. Glial vacuoles are frequently seen in the retina of null mutant flies, and asterisks mark vacuoles in glia, which are rarely observed in y^1^ w^*^ flies. (**B**) TEM images of fly retina of the indicated genotypes upon 7 days of D/L cycles. The overall morphology of the retina is severely affected in y^1^ w*; Gba1b^T2A-Gal4^/Df flies, whereas the retinas of y^1^ w^*^ flies do not show obvious defects. The number of intact photoreceptors (PRs) per ommatidium is quantified on the right. Error bars represent SEM (*n* = 3); ****P < 0.0001. Flies that are tested in this experiment are phenotypically white.

To assess ultrastructural defects in the fly retinas, we performed transmission electronic microscopy (TEM) at days 2 and 7 for flies kept in D/L cycles. All flies used in these experiments were in a *white^−^* background (y^1^ w*; Gba1b^T2A-GAL4^/Df or y^1^ w* control). After 2 days of D/L exposure, glia in the *Gba1b* null mutants exhibit large vacuoles (~1.5 μm^2^) that are not or rarely observed in y^1^ w^*^ control animals [Fig. 3A (b and b′, white asterisks) and fig. S3A]. Glia in y^1^ w^*^ flies are tightly associated with photoreceptor neurons (Fig. 3A, a and a′, highlighted in blue), whereas many glia in mutant retina are detached from the photoreceptor neurons [Fig. 3A (b and b′, magenta arrow) and fig. S3B]. We also observe a vast increase in the number of lysosomes in glia of mutant animals when compared to y^1^ w^*^ flies at day 2 [Fig. 3A (b and b′, red arrowheads) and fig. S3C]. In contrast, we did not observe obvious morphological defects in photoreceptor neurons, nor did we observe an increase in lysosomes in these cells (Fig. 3A and fig. S3C). These data show that the primary morphological lesions originate in glia.

As shown in Fig. 3B, TEM after 7 days of D/L cycles shows that the photoreceptor morphology is severely affected in *Gba1b* null mutants (Fig. 3B, b), consistent with the severe ERG defects documented in Fig. 2C. In contrast, the neurons and glia in y^1^ w^*^controls show no or very minor defects at this stage (Fig. 3B, a). The data clearly show that loss of *Gba1b* causes defects in glia that precede the defects in photoreceptor neurons. Moreover, *Gba1b* is required for neuronal maintenance.

### Loss of *Gba1b* causes light/activity-dependent GlcCer accumulation in neurons and glia

**Fig. 4.**
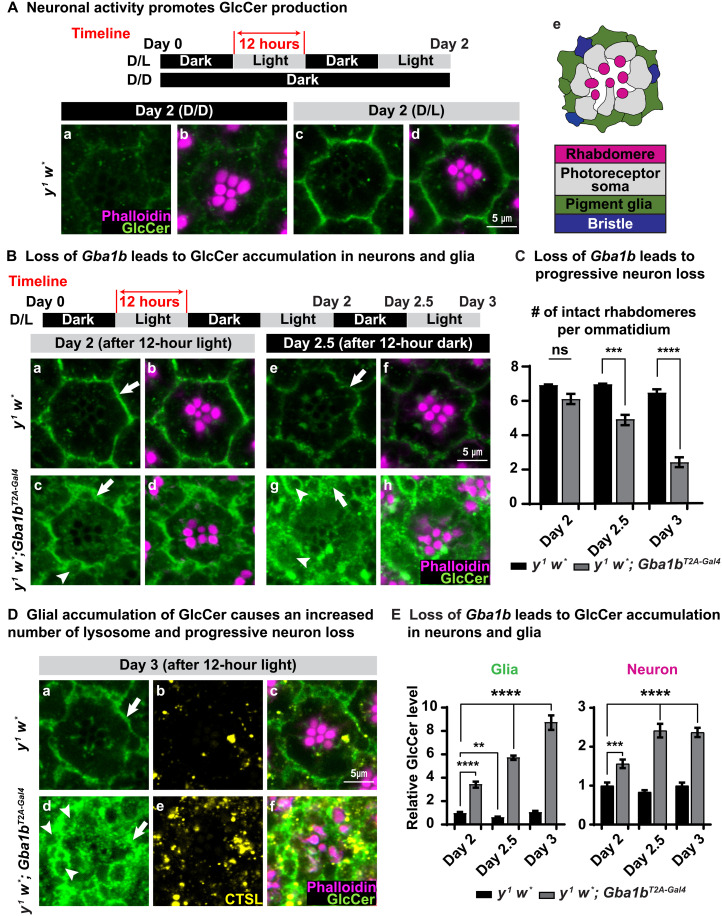
Loss of *Gba1b* leads to GlcCer accumulation. (**A**) Immunofluorescent images of fly retina of the indicated genotypes and the conditions under which the flies were raised: antibody against GlcCer in green, whereas phalloidin labels rhabdomeres in magenta. (a and b) Flies kept in the dark show very little GlcCer in the ommatidia of y^1^ w^*^ (basal level). (c and d) After 12 hours of light exposure, GlcCer is synthesized and accumulates in retina of *y w* flies (*n* ≥ 9). (e) A cartoon image illustrating the structure of a fly ommatidium. (**B**) (a, b, e, and f) GlcCer accumulation in the glia of y^1^ w^*^ flies is reduced after 12 hours of inactivation of neurons in the darkness. (a to d) After 2 days of D/L cycles, similar to *y w* flies, GlcCer accumulates in the glia of y^1^ w*; Gba1b^T2A-Gal4^ flies. However, GlcCer accumulates more in the neurons of y^1^ w*; Gba1b^T2A-Gal4^ flies. (c, d, g, and h) The glial accumulation of GlcCer fails to be degraded upon exposure to 12 hours of darkness in retina of y^1^ w*; Gba1b^T2A-Gal4^ flies (*n* ≥ 9). (**C**) Loss of *Gba1b* leads to progressive neurodegeneration. Numbers of intact PRs per ommatidium in (B) and (D) were quantified. Error bars represent SEM (*n* ≥ 9); ***P < 0.001 and ****P < 0.0001. (**D**) (a, c, d, and f) GlcCer progressively accumulates in the retina of y^1^ w*; Gba1b^T2A-Gal4^ flies, which is highly enriched in the glial region. (b and e) CTSL (yellow) represents lysosomes. The number of lysosomes is increased in y^1^ w*; Gba1b^T2A-Gal4^ flies compared with y^1^ w^*^ flies (*n* ≥ 9). (**E**) Quantification of relative GlcCer levels in (B) and (D). Error bars represent SEM (*n* ≥ 9); **P < 0.01, ***P < 0.001, and ****P < 0.0001. All flies that are tested in this experiment are phenotypically white.

To determine how loss of *Gba1b* leads to functional (Fig. 2 and fig. S2) and morphological (Fig. 3) defects in the nervous system, we measured the distribution and levels of GlcCer, the substrate of Gba1b protein. As shown in Fig. 4A (a and b) and fig. S4A (a and b), both y^1^ w^*^ and y^1^ w*; Gba1b^T2A-Gal4^ (homozygous y^1^ w*; Gba1b^T2A-Gal4/T2A-Gal4^ alleles are labeled y^1^ w*; Gba1b^T2A-Gal4^ hereafter) flies that are kept in constant darkness (D/D) do not show obvious GlcCer signals based on immunostaining (the green signals in Fig. 4A and fig. S4A). Upon 12 hours of light stimulation, we observed GlcCer accumulation in neurons and glia in the retinas of both y^1^ w^*^ and y^1^ w*; Gba1b^T2A-Gal4^ flies [Fig. 4A (c and d) and fig. S4A (c and d)]. Hence, light-induced activity of photoreceptor neurons promotes the synthesis of GlcCer.

To determine whether neural activity triggers GlcCer synthesis besides the visual system, we overexpressed *trpA1* in fly neurons driven by *elav-GAL4* (*45*). *trpA1* encodes a thermosensitive transient receptor potential channel (TRP channel). It is a cation channel that is not permeable to Ca^2+^ and Na^+^ ions at 18°C but is highly permeable to these ions at 29°C (*46*). Transgenic flies (e*lav-GAL4* >*dTrpA1*) and negative control flies (e*lav-GAL4* >*empty*) were raised at 18°C till 3 days after eclosion. They were then exposed at 29°C for 60 min to promote neuronal activity (fig. S4B). We performed immunostaining on paraffin brain sections using the GlcCer antibody. As shown in fig. S4B, a significant increase in GlcCer levels was observed in many brain areas of the fly that overexpressed *dTrpA1* in the neurons, indicating that neuronal activity promotes GlcCer synthesis in the CNS.

To further assess the effect of light exposure on GlcCer accumulation and neurodegeneration, we exposed the y^1^ w^*^ and y^1^ w*; Gba1b^T2A-Gal4^ flies for 12 hours to light [day 2; Fig. 4, B (a to d) and C] and then transferred them into the dark for 12 hours [day 2.5; Fig. 4, B (e to h) and C]. As shown in Fig. 4B (e and f), compared with Fig. 4B (a and b), the GlcCer levels in the y^1^ w^*^ control flies are reduced upon dark exposure. In contrast, when compared with Fig. 4B (c and d), the levels of GlcCer in the y^1^ w*; Gba1b^T2A-Gal4^ flies are not reduced but rather accumulate in glia and photoreceptor neurons (Fig. 4B, g and h) upon dark exposure. Furthermore, the accumulation of GlcCer is exacerbated when the y^1^ w*; Gba1b^T2A-Gal4^ flies were further kept for a second period of 12 hours in light (day 3; Fig. 4D, a, c, d, and f). Moreover, we observed a robust expansion in size of the pigment glia (white arrows in Fig. 4, B and D) and an increase in vacuoles in these glia (white arrowheads in Fig. 4, B and D). To monitor the morphology of photoreceptor neurons, we used phalloidin to label the rhabdomeres (*47*). We do not observe neurodegeneration at day 2 [phalloidin in Fig. 4, B (b and d) and C]. This is consistent with previous TEM data at day 2 (Fig. 3A). However, we observe a progressive loss of photoreceptor neurons in the mutants at day 2.5 [phalloidin in Fig. 4, B (f and h) and C] and day 3 [phalloidin in Fig. 4, D (c and f) and C]. Note that loss of *Gba1b* causes morphological defects in the pigment glia before neurodegeneration, consistent with our TEM data (Fig. 3). In summary, the data show that light stimulation promotes the elevation of GlcCer in both neurons and pigment glia, and loss of *Gba1b* causes severe morphological defects in pigment glia followed by the loss of the photoreceptor neurons.

### GlcCer transport to glia for lysosomal degradation by Gba1b

To explore how GlcCer accumulates in glia, we measured the relative elevation of GlcCer intensity in y^1^ w^*^ and y^1^ w*; Gba1b^T2A-Gal4^ flies raised under different light stimulation paradigms (Fig. 4E). Upon light stimulation at day 2, we found that GlcCer accumulates more in both neurons and glia of y^1^ w*; Gba1b^T2A-Gal4^ mutants than in y^1^ w^*^ flies (first two columns in the neuron and glia plots of Fig. 4E). When we compared the GlcCer levels between pigment glia of y^1^ w^*^ flies at days 2, 2.5, and 3, we observed a fluctuation in levels that correlates with light exposure. This fluctuation of GlcCer in the pigment glia of y^1^ w^*^ flies is consistent with the model that light/activity stimulates an increase in GlcCer levels, and darkness permits elevated GlcCer levels to return to a lower baseline (black columns in the glia plot of Fig. 4E). In contrast, in the absence of this light/activity, there is an accumulation with time in y^1^ w*; Gba1b^T2A-Gal4^ (gray columns in the glia plot of Fig. 4E). These data suggest that GlcCer is produced in active neurons and accumulates in both neurons and glia when *Gba1b* is lost.

Given that *Gba1b* is a lysosomal hydrolase, we assessed the presence of lysosomes in y^1^ w^*^ and y^1^ w*; Gba1b^T2A-Gal4^ flies at day 3 using immunofluorescence staining of Cathepsin L (CTSL), a lysosomal enzyme (Fig. 4D, b, c, e, and f) (*48*). The y^1^ w*; Gba1b^T2A-Gal4^ mutant ommatidia show an increased number of lysosomes when compared with y^1^ w^*^ (fig. S4D). Most of these lysosomes are localized in glia (Fig. 4D, b, c, e, and f), indicating that loss of *Gba1b* leads to a progressive glial GlcCer accumulation that impairs glial lysosomes.

**Fig. 5.**
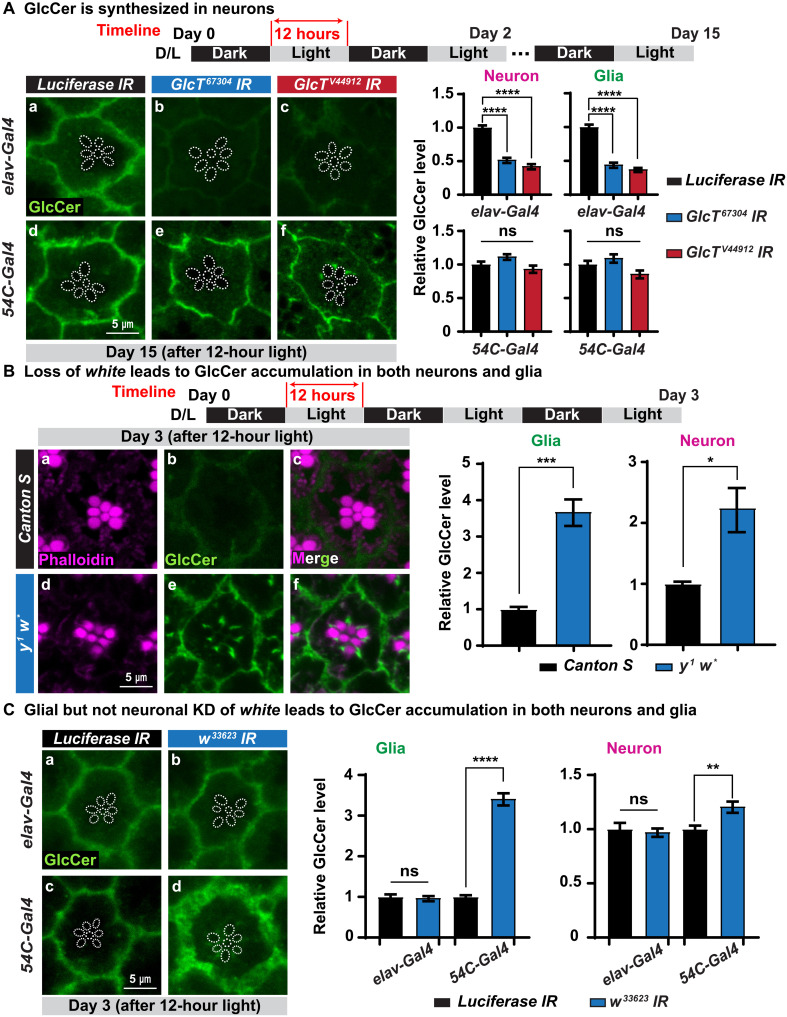
Loss of *white* in glia causes GlcCer accumulation. (**A**) GlcCer is produced in neurons. Immunofluorescent images of fly ommatidia of the indicated genotypes after 15 days of D/L cycles. *Glucosylceramide synthase* (*GlcT*) was knocked down using two different RNAi constructs in neurons driven by *elav-Gal4* (top) and in glia driven by *54C-Gal4* (bottom), respectively. Neuronal but not glial knockdown of *GlcT* leads to a reduction of GlcCer in both neurons and glia. Dashed circles outline rhabdomeres. Relative GlcCer levels are quantified on the right. Error bars represent SEM (*n* ≥ 11), ****P < 0.0001. Flies that are tested in this experiment are red eyed because of the transgene. (**B**) Loss of *white* causes GlcCer accumulation. Immunofluorescent images of fly retina of the indicated genotypes after 3 days of D/L cycles. GlcCer accumulates in the ommatidia of *y w* but not in *Canton S* flies. Relative GlcCer levels are shown on the right. Error bars represent SEM (*n* ≥ 7), *P < 0.05 and ***P < 0.001. (**C**) Glial but not neuronal knockdown (KD) of *white* causes GlcCer accumulation. Immunofluorescent images of fly retina of the indicated genotypes after 3 days of D/L cycles. The *white* mRNA was knocked down using a *UAS-RNAi* construct expressed in neurons by *elav-Gal4* (top) and in glia driven by *54C-Gal4* (bottom), respectively. Knockdown of *white* in glia leads to an accumulation of GlcCer in both neurons and glia, whereas neuronal knockdown in neurons does not cause a phenotype. Relative GlcCer levels are quantified on the right. Error bars represent SEM (*n* = 11), **P < 0.01 and ****P < 0.0001. Flies that are tested in this experiment are red eyed because of the transgenes.

As GlcCer is produced upon neuronal activity and progressively accumulates in glia of y^1^ w*; Gba1b^T2A-Gal4^ mutants, we hypothesized that GlcCer is synthesized in neurons and subsequently transported to glia for lysosomal degradation by Gba1b. To test this hypothesis, we knocked down *Glucosylceramide synthase* (*GlcT*), the gene encoding the ceramide glucosyltransferase that produces GlcCer, either in neurons (driven by *elav-Gal4*) or in pigment glia (driven by *54C-Gal4*) in wild-type flies. Neuronal (Fig. 5A, a to c) but not glial (Fig. 5A, d to f) knockdown of *GlcT* significantly reduces GlcCer levels, suggesting that GlcCer is indeed produced in neurons. The latest fly single-cell sequencing database shows that *GlcT* is highly expressed in neurons including the photoreceptor neurons (*49*).

In summary, our data show that GlcCer is synthesized in neurons by GlcT upon light stimulation and is somehow transported to pigment glia for lysosomal degradation by Gba1b. Upon loss of *Gba1b*, GlcCer accumulates in the photoreceptor neurons and pigment glia, causing glial expansion and vacuoles in glia. This accumulation is toxic to both cell types, although the glial impairment precedes neuronal loss.

### Loss of *white* exacerbates GlcCer accumulation and neurodegeneration

The y^1^ w*; Gba1b^T2A-Gal4^ allele was generated in a *white^−^* background using *3XP3-GFP* as a dominant marker (*32*), whereas the *Gba1b^STOP/STOP^* and all RNAi lines carry the *mini-white* as a dominant marker. As documented before (Fig. 2, B and C, and fig. S2B), y^1^ w*; Gba1b^T2A-Gal4^ mutants exhibit defects much earlier than *Gba1b^STOP/STOP^* mutants, while both alleles are thought to be strong loss-of-function alleles (*22*). The *white* gene encodes an ABC [adenosine 5′-triphosphate (ATP)–binding cassette] transporter that is known to transport molecules that make up the pigment granule, a lysosomal-like organelle (*30*, *31*). The fly White protein is conserved with human ABCG family transporters that are known to be lipid transporters (*50*). Considering that *white* has been found to affect light-induced photoreceptor degeneration and life span (*51*) similar to *Gba1b*, we assessed whether the *white* gene might affect GlcCer trafficking and participate in GlcCer metabolism.

To explore the impact of *white* on GlcCer metabolism and neurodegeneration, we performed immunostaining for GlcCer and compared its levels in neurons and glia of *Canton S* (*white^+^*) and y^1^ w^*^ (*white^−^*) flies at day 3. As shown in Fig. 5B, GlcCer levels are significantly increased in both neurons and glia of y^1^ w^*^ flies when compared with *Canton S* flies. Hence, loss of *white* is sufficient to cause GlcCer accumulation.

The White protein has been shown to be abundant in pigment glia and present at much lower levels in photoreceptor neurons (*30*, *31*). Unfortunately, the antibody used to perform these studies is no longer available. To determine which type of cells requires the White protein, we knocked down the *white* gene in glia driven by *54C-Gal4* and in neurons using *elav-Gal4*. As shown in fig. S5A, neuronal knockdown does not cause an obvious alteration in pigmentation, whereas glial knockdown reduces or abolishes pigmentation in females and males, respectively. GlcCer does not accumulate in glia or neurons when *white* is knocked down in neuron (Fig. 5C, a and b). However, glial knockdown leads to an accumulation of GlcCer and an expansion of the glial cells (Fig. 5C, c and d). The data show that loss of *white* in glial cells impairs GlcCer degradation.

To assess how White may modulate GlcCer trafficking, we first determined the subcellular localization of ectopic expressed White protein in *Drosophila* Schneider 2 (S2) cells. As shown in fig. S5B, White::HA is colocalized with the multivesicular body (MVB) marker hepatocyte growth factor–regulated tyrosine kinase substrate (Hrs) (*52*) (fig. S5B, m to o), but not mitochondrial (Atp5a; fig. S5B, a to c), lysosomal (CTSL; fig. S5B, d to f), early endosomal (Rab5; fig. S5B, g to i), or late endosomal (Rab7; fig. S5B, j to l) markers. This indicates that White may modulate GlcCer trafficking by regulating the endolysosomal pathway in MVBs. We then exposed the S2 cells to Biotin-GlcCer and determined the White::HA subcellular localization (fig. S5C). White-HA remains associated with MVBs upon Biotin-GlcCer exposure (fig. S5C, a to f), but a substantial fraction of White::HA becomes colocalized with the lysosomal marker CTSL (fig. S5C, g to l), suggesting that White is associated with MVB that traffics to lysosomes upon GlcCer exposure.

### GlcCer is transported from neurons to glia in mammalian cell coculture assays

**Fig. 6.**
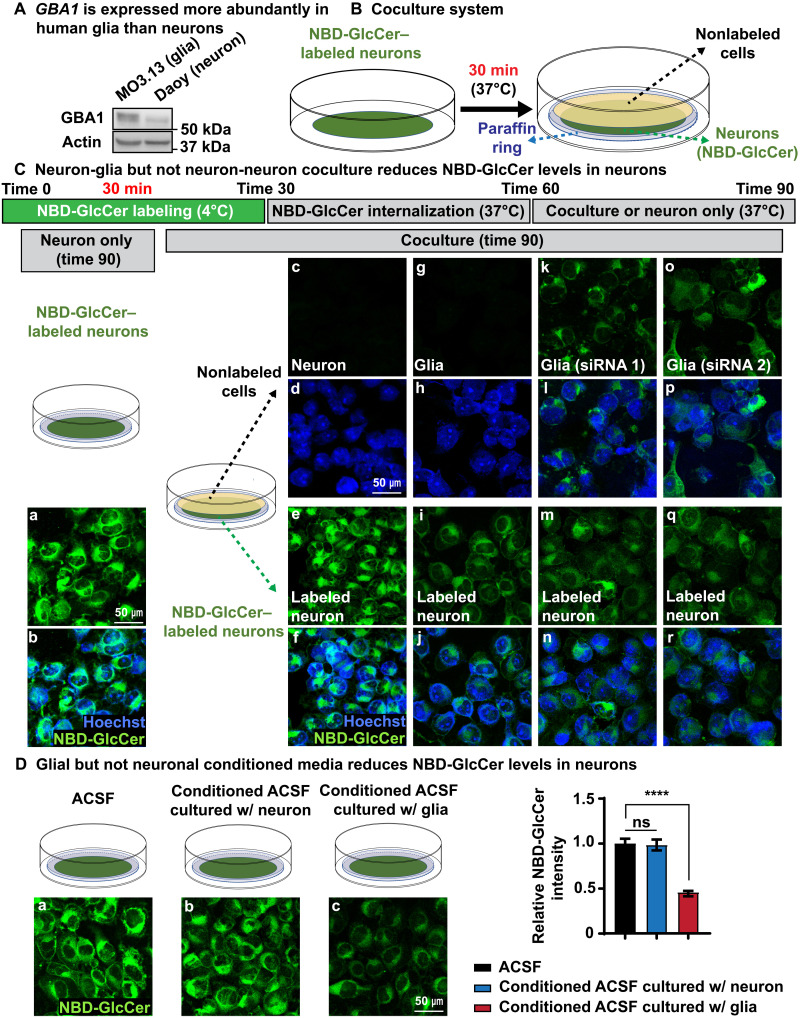
Neuron-to-glia GlcCer transport is evolutionarily conserved in human cells. (**A**) A human oligodendrocyte cell line, MO3.13, expresses more *GBA1* than human Daoy neurons. (**B**) A cartoon illustrating the coculture system. Daoy cells are labeled with C6-NBD-GlcCer (green, NBD-GlcCer). A paraffin ring is then placed on top of the labeled neuron. Other cells, Daoy neurons, or glia are cultured on a coverslip. The latter are inverted so that they bathe in the same fluid as the labeled neurons. Hence, the two groups of cells face each other but are separated by the paraffin ring. (**C**) (a and b) After labeling at 4°C and 30 min at 37°C, NBD-GlcCer is partially internalized into the cytosol of labeled neurons. They are then cultured in ACSF for an additional 30 min. The NBD-GlcCer remains in the cells for the entire period. (c to f) Neuron-neuron coculture does not reduce the NBD-GlcCer levels in labeled neurons. Little to no NBD-GlcCer signal is detected in nonlabeled neurons. (g to j) Neuron-glia coculture leads to a decrease in NBD-GlcCer levels in labeled neurons. A faint signal is observed in the nonlabeled MO3.13 glia (g). (k to r) Labeled neurons are cocultured with nonlabeled MO3.13 glia in which *GBA1* is knocked down using two different siRNAs. Again, the NBD-GlcCer level is reduced in the labeled neurons under (*GBA1* knocked down) coculture conditions. NBD-GlcCer is retained in the nonlabeled MO3.13 glia. (**D**) Glial but not neuronal conditional medium promotes NBD-GlcCer release from labeled neurons. NBD-GlcCer–labeled neurons were incubated with three different conditional ACSF media as shown in (a) to (c) for 30 min, respectively. Only ACSF that was used to culture glia (c) is able to trigger NBD-GlcCer release from labeled neurons. Relative NBD-GlcCer intensity is quantified on the right. ****P < 0.0001.

Our data in flies suggest that *Gba1b* plays an important role in GlcCer metabolism in glia. Human transcriptome data show that human *GBA1* is expressed at higher levels in astrocytes than in neurons (*53*). The Brain RNA-Seq database (Brain RNA-Seq) shows that *Gba*, the mouse homolog of *GBA1*, is expressed more in microglia, oligodendrocytes, and astrocytes than in neurons (*54*). These results are consistent with a potential role of glia in GlcCer metabolism in our fly model. We therefore compared human GBA1 protein levels in a human medulloblastoma cell line with neuronal properties (Daoy cells; we will call them neurons hereafter) (*55*) and a human oligodendrocyte cell line (MO3.13 oligodendrocytes; we will call them glia hereafter) (*56*). The level of GBA1 is indeed significantly higher in these human glial cells than in Daoy neurons (Fig. 6A). We then established a coculture system to test whether the transport of GlcCer from neurons to glia occurs in mammalian cells and assess whether the role of *GBA1* in glia during this process is evolutionarily conserved.

To label the GlcCer, we incubated Daoy neurons with fluorescent C6-nitrobenzoxadiazole–tagged GlcCer (NBD-GlcCer for short) for 30 min at 4°C. This labels the plasma membrane of neurons (fig. S6A, a and b). Upon shifting the cells to 37°C for 30 min, the NBD-GlcCer is partially internalized into the cytosol (time 60; fig. S6A, c and d), and a similar signal is observed 30 min later (time 90; fig. S6A, e to g). To monitor whether NBD-GlcCer can be transferred to other cells, the labeled Daoy neurons were cocultured with four different types of cells: Daoy neurons, MO3.13 glia, and two independently derived MO3.13 glial cells in which *GBA1* was knocked down by small interfering RNAs (siRNAs) (fig. S6C). In these assays, the labeled neurons are placed at the bottom, whereas the cell layer of the four different cell types is cultured on a coverslip that is placed above. The top layer of cells does not come into direct contact with the cells at the bottom as a ring of paraffin film separates both cell layers. Labeled neurons that were not cocultured with other cells (Fig. 6C, a and b) or labeled neurons that are overlaid with nonlabeled neurons do not show a reduction in NBD-GlcCer levels after 30 min [Fig. 6C (e and f) and fig. S6B]. Also, no to little NBD-GlcCer signal is detected in the nonlabeled neurons placed on top in this assay (Fig. 6C, c and d), indicating that NBD-GlcCer is not transported between neurons. However, when the labeled neurons were cocultured with glia, we observed a significant reduction of the levels of NBD-GlcCer in the labeled neurons [Fig. 6C (i and j) and fig. S6B]. Moreover, only a very faint NBD-GlcCer signal is now detected in glia. This suggests that glial cells trigger the transport of NBD-GlcCer from labeled neurons and that they may be able to uptake GlcCer for degradation [Fig. 6C (g and h) and fig. S6B].

If glial cells are able to take up the NBD-GlcCer released from labeled neurons, reduction of GBA1 in glia should lead to an accumulation of GlcCer. We therefore knocked down *GBA1* in glia using two independent siRNAs (fig. S6C). We then overlaid the labeled neuronal cells with these glial cells to determine whether they accumulate GlcCer that is taken up from the medium. As shown in Fig. 6C, we observed a robust accumulation of NBD-GlcCer in these glial cells [Fig. 6C (k, l, o, and p) and fig. S6D], providing compelling evidence that NBD-GlcCer from labeled neurons is indeed transported to glia and that *GBA1* plays a role in the degradation process. In summary, these data show that GlcCer is transported from neurons to glia and that this process is evolutionally conserved between flies and human cells.

### TGF-β/BMP promotes the transfer of GlcCer from neurons to glia via exosomes

Given that GlcCer is transported from neurons to glia but not between neurons, we hypothesized that glia may release a signal that stimulates neurons to secrete GlcCer. We therefore collected conditioned medium from neuronal and glial cell cultures. When NBD-GlcCer–labeled neurons were exposed to conditioned artificial cerebrospinal fluid (ACSF) from neurons for 30 min, no obvious changes were observed at NBD-GlcCer levels (Fig. 6D, a and b). In contrast, conditioned ACSF medium from glia induced a severe reduction in NBD-GlcCer signal in labeled neurons (Fig. 6D, a and c). These data indicate that glia secrete a factor that induces the release NBD-GlcCer from labeled neurons.

**Fig. 7.**
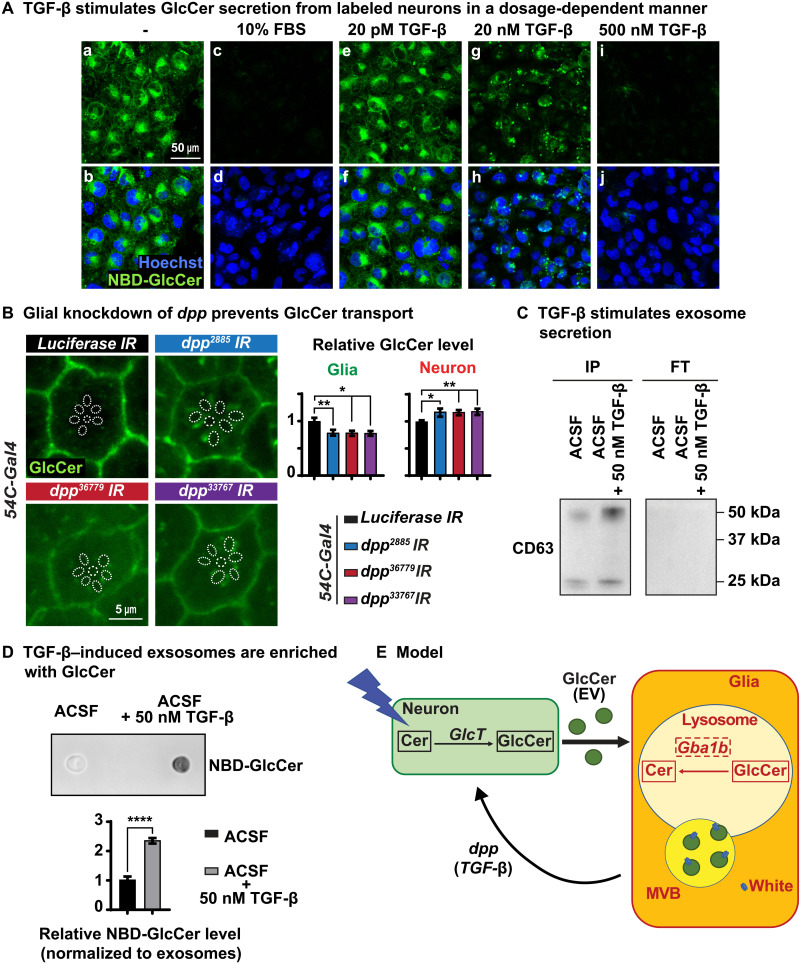
TGF-β triggers GlcCer secretion in the form of exosomes. (**A**) Growth factor TGF-β stimulates the release of NBD-GlcCer of labeled neurons. NBD-GlcCer–labeled neurons were incubated with five different conditional ACSF media as indicated for 30 min, respectively. (a to d) ACSF with 10% FBS promotes the complete release of NBD-GlcCer from labeled neurons. (a, b, and e to j) ACSF with TGF-β triggers the release of NBD-GlcCer from labeled neurons in a dosage-dependent manner. (**B**) Glial knockdown of *dpp* causes elevated GlcCer in the photoreceptor neurons and reduced GlcCer level in the pigment glia. Relative GlcCer intensity is quantified on the right. Error bars represent SEM (*n* = 11), *P < 0.05 and **P < 0.01. (**C**) NBD-GlcCer–labeled neurons were incubated with two conditional ACSF media: ACSF only and ACSF with 50 nM TGF-β for 1 hour. Immunoprecipitation (IP) of CD63-positive exosomes from two conditional ACSF media as indicated. FT, flow-through. ACSF with 50 nM TGF-β contains significantly more exosomes from NBD-GlcCer–labeled neurons compared with ACSF only. (**D**) Exosomes released from NBD-GlcCer–labeled neurons are enriched with GlcCer. A dot blot was performed to determine the levels of NBD-GlcCer in exosomes isolated from the two conditional ACSF media in (C). Hence, ACSF with 50 nM TGF-β contains many more exosomes, and these exosomes are enriched with NBD-GlcCer. (**E**) Working model. GlcCer is synthesized in neurons upon neuronal activity. It is then transported to glia via exosomes for lysosomal degradation by *Gba1b*. TGF-β/Dpp secreted from glia is sufficient to stimulate neuronal release of exosomes, which are enriched with GlcCer. *white* (ABCG) plays a major role in glia in the degradation of GlcCer and is associated with MVBs, and loss of both *white* and *Gba1b* synergizes and exacerbates the accumulation of GlcCer, suggesting that both participate independently in the degradation of GlcCer.

In all previous experiments, we used a defined medium: ACSF, which contains salts and glucose but no serum or growth factors. To explore whether cell signaling molecules present in other media are able to promote the release of GlcCer from neurons, we added 10% fetal bovine serum (FBS) to ACSF. To our surprise, ACSF containing 10% FBS led to a near-complete release of NBD-GlcCer from labeled neurons upon a 30-min exposure (Fig. 7A, a to d), suggesting that FBS contains key factors that can promote the release of NBD-GlcCer from neurons. FBS contains various growth factors including Insulin-like growth factor–binding protein 2 (*IGFBP2*), Transforming growth factor–β (*TGF-*β), and Glial growth factor (*GGF*), among other growth factors (*57*). In the *Drosophila* visual system, the cone and pigment cells that function as glial cells (*39*, *40*) express elevated levels of *dpp* and *daw*, two genes encoding ligands of the TGF-β/BMP superfamily (fig. S7A) (*49*). Moreover, it has been reported that mammalian glia produce TGF-β1, which activates TGF-β signaling in adult mouse neurons (*58*). We therefore explored whether TGF-β can promote the transfer of GlcCer from neurons to glia.

We first tested whether human TGF-β was able to trigger the release of GlcCer from labeled neurons in our cell culture system. TGF-β (20 pM) did not affect the release of NBD-GlcCer (Fig. 7A, e and f), whereas 500 nM TGF-β led to a near-complete loss of NBD-GlcCer from labeled neurons (Fig. 7A, i and j). At lower doses of 20 nM, a dose in the physiological range (*59*), we observe a redistribution and a reduction of NBD-GlcCer in neurons (Fig. 7A, g and h). Note that these assays were performed after a 30-min exposure. Hence, these data show that the addition of TGF-β is sufficient to promote the release of GlcCer from labeled neurons in a short time frame.

To determine whether glial TGF-β signaling can stimulate GlcCer secretion in flies, we specifically reduced the level of expression of *dpp* in the pigment glia using three different RNAi lines including a documented RNAi line (*60*). As shown in Fig. 7B, glial knockdown of *dpp* causes a mild but significant elevation of GlcCer in photoreceptor neurons as well as a mild but significant reduction of GlcCer level in pigment glia. These data suggest that a glial-expressed *dpp* is involved in GlcCer release from neurons in *Drosophila*. In summary, the data indicate that TGF-β/BMP signals produced in glia promote the release of GlcCer produced in neurons.

To determine the mechanism by which GlcCer is transported from neurons to glia, we first explored the role of two apolipoproteins encoded by *Neuronal Lazarillo* and *Glial Lazarillo* (*39*, *61*). We previously showed that *Glial Lazarillo* plays a critical role in the transport of neutral lipids from neurons to glia in the fly visual system (*61*). However, as shown in fig. S7B, a severe loss of these proteins induced by RNAi did not affect the levels of GlcCer in neurons and glia, suggesting that GlcCer transport from neurons to glia may not rely on lipoprotein particles.

Previous work has shown increased numbers of extracellular vesicles (EVs) in heads of *Gba1b* mutant flies (*62*), suggesting that increased levels of GlcCer lead to an increase in EVs. Moreover, Corrigan *et al.* (*63*) presented compelling data that *Drosophila* male reproductive glands secrete exosomes under the regulation of BMPs, members of TGF-β superfamily. Hence, GlcCer may be transported from neurons to glia via exosomes. To determine whether this is the case, we isolated CD63-positive exosomes (*64*) from ACSF media that were used to culture NBD-GlcCer–labeled neurons. As shown in Fig. 7C, Western blots show an increase in CD63-positive exosomes in ACSF upon the addition of 50 nM TGF-β when compared to ACSF without TGF-β. Dot blots of these CD63-positive exosomes exposed to TGF-β are highly enriched in NBD-GlcCer (Fig. 7D). Together, these data support the idea that TGF-β triggers GlcCer release from neurons to glia via exosomes.

To determine whether TGF-β promotes GlcCer-enriched exosome secretion, we purified exosomes from media of cells that were treated with and without 50 nM TGF-β for 1 hour and performed TEM. TEM images showed that vesicles purified from media of neurons that were treated with TGF-β have a diameter of around 80 nm (fig. S7C, b). However, no vesicles were detected from media of neurons that were not treated with TGF-β (fig. S7C, a). These data further support the notion that TGF-β triggers exosome secretion. Using the same experimental paradigm, we performed lipidomics on the neuronal media. As shown in fig. S7D, we observed significantly increased levels of ceramide and GlcCer in the cell culture media treated with TGF-β when compared to the nontreated cell culture media, indicating that TGF-β promotes ceramide and GlcCer secretion. No significant difference was observed in the cell pellets for ceramide and GlcCer, although they showed a consistent decrease for both (fig. S7D). In the context of the observations with the CD63-positive vesicles and the TEM data, we conclude that GlcCer-containing exosomes are secreted upon a TGF-β signal.

Given that exosomes are released by the fusion of MVBs with the plasma membrane and given that large MVBs are often observed by TEM in *Drosophila* photoreceptors, we performed RNAi experiments to reduce the levels of three ESCORT complex proteins in photoreceptor neurons: *CHMP2B*, *Mvb12*, and *lsn*. We assessed the levels of GlcCer in glia and observed no accumulation of GlcCer after 15 days of D/L cycles (fig. S7E). Hence, these data are consistent with the above observations that GlcCer is transported via exosomes from neurons to glia. Note that the levels of GlcCer in neurons are also decreased when we reduced the levels of these ESCORT complex proteins specifically in neurons. This is also consistent with the previous observations that ESCORT complex affects GlcCer synthesis (*65*, *66*).

## DISCUSSION

Here, we show that GlcCer, the substrate of *Gba1b* in flies and *GBA1* in humans, is produced in neurons upon neuronal activity in flies and is transported from neurons to glia via exosomes. Upon exposure to TGF-β/BMP signals produced in glia, GlcCer-enriched exosomes are released from neurons, and these exosomes are taken up by glia. We propose that GlcCer is then endocytosed by glia where it is degraded in the lysosomes through the action of *Gba1b/GBA1*. We also show that *Drosophila Gba1b* is expressed in glia in the CNS during all developmental stages as well as in adults. Similarly, GBA1 protein levels are also much higher in human glial cells than neurons. The ABCG transporter, White, plays an important role in the degradation of GlcCer in pigment glia in flies and is present in MVBs (Fig. 7E).

### *Gba1b* is necessary and sufficient in *Drosophila* glia to support neuronal function

We show that *Gba1b* is required in glia to support neuronal function and that the human *GBA1*, when expressed in glial cells, rescues the null mutant phenotype. However, the mutant *GBA1^N370S^* variant associated with GD and PD (*20*) fails to rescue *Gba1b* mutant phenotypes (*21*). Our data argue that expression of *Gba1b* in glia is necessary and sufficient to maintain proper neuronal activity.

Ultrastructural TEM images demonstrate that loss of *Gba1b* leads to impaired and vacuolized pigment glia before neuronal degeneration. These glial cells accumulate elevated levels of GlcCer and exhibit lysosomal expansion based on costaining with the lysosomal marker CTSL. Glial GlcCer accumulation impairs the morphology of glia first and is followed by the demise of neurons. Although most studies on *GBA1* and GD focus on defects in neurons, a study by Keatinge *et al.* (*24*) reported that activation of microglia precedes neuronal demise in zebrafish. Knocking out *Gba* in both neurons and glia of mice driven by *Nestin-Cre* leads to paralysis 21 days after birth (*27*), whereas a knockdown of *Gba* in mice dopaminergic neurons causes robust microglial activation but does not affect motor behavior and does not cause dopaminergic neuron loss (*67*). These results again suggest that GBA1 may play a more important role in glia than in neurons in the pathogenesis of neurodegenerative diseases. However, we show that human GBA1 (Fig. 6) is present in neurons at relatively low levels when compared to glia. In addition, neuronal functions of GBA1 in some neurons have previously been reported (*68*–*70*). However, our data and these vertebrate studies show that GBA1 is present in glial cells as well (*68*–*70*). In summary, our data show that *Gba1b* plays an essential role in glia and is required for the maintenance of neurons in *Drosophila*.

### Neuronal activity promotes GlcCer production

UDP-glucose:ceramide glucosyltransferase (*UGCG*, *GlcT* in flies) catalyzes the synthesis of GlcCer, and its loss leads to embryonic lethality (*71*). The conditional and selective removal of *Ugcg* in mouse neurons and glia causes severe neural abnormalities after birth and lethality at weaning (*72*). The synthesis of GlcCer has been shown to be triggered by endotoxin lipopolysaccharides as they induce *Ugcg* mRNA transcription (*73*). Increased levels of *Ugcg* mRNA have been shown in tumor cells (*74*). However, GlcCer synthesis has not been linked to neuronal activity to our knowledge. Our data show that y^1^ w^*^ or y^1^ w*; Gba1b^T2A-Gal4^ mutant flies that are kept in the dark do not accumulate GlcCer in photoreceptor neurons or pigment glia. However, upon light exposure, y^1^ w^*^ ommatidia show an accumulation of GlcCer in neurons and glia, but the accumulation of GlcCer is obviously greater in y^1^ w*; Gba1b^T2A-Gal4^ than in y^1^ w* ommatidia. When we trigger neuronal activity by activating *Drosophila* TrpA1 channel in the fly brain for 60 min, we observe a significant increase in GlcCer levels in most areas of the fly brain. Hence, these data show that neuronal activity promotes the production of GlcCer. Our data strongly indicate that neuronal activity promotes GlcCer synthesis. However, they do not exclude the possibility that neuronal activity inhibits GlcCer degradation.

The loss of the synthesis of GlcCer in glia, unlike the loss of GlcCer in neurons, is not associated with a phenotype. Similarly, loss of *Ugcg* in oligodendrocytes does not cause obvious phenotypes in mice (*75*), suggesting that GlcCer is produced by other cells and transported to glia (*76*). Consistently, we find that loss of *GlcT*, the fly homolog of *UGCG*, in glia does not affect the levels of GlcCer, whereas loss of *GlcT* in neurons strongly reduced the levels of GlcCer in neurons and glia. Neuronal activity leads to an increased level of GlcCer in neurons and glia. However, how the transport of GlcCer is regulated and what triggers the transport are unknown.

### Glia secrete a TGF-β/BMP signal to promote the release of GlcCer-enriched exosomes

We found that only neuron-glia but not neuron-neuron coculture stimulates NBD-GlcCer release from labeled neurons. Moreover, conditional ACSF from glia but not from neurons promotes NBD-GlcCer release from neurons. These results implicate that molecular cues that are secreted from glia trigger GlcCer release. Our data show that TGF-β is sufficient to promote the release of NBD-GlcCer from neurons. After exposing the labeled neurons to TGF-β, we observed a very significant increase in CD63-positive exosomes and an increase in NBD-GlcCer in the exosomal fraction. Similarly, when neurons are not labeled but stimulated with TGF-β, we observe an increase in GlcCer in the culture media based on lipidomic assays. Given that lysosomal enzymes have been previously shown to be shared among nearby cells (*77*) and that Gba1b can be transported between cells via exosomes in flies (*78*), we also assessed whether GBA1 was present in the media or the exosomes produced by the neurons. However, we did not detect GBA1 proteins in exosomes or culture media. Hence, a significant fraction of neuronal GlcCer must be transported through exosomes to glia upon TGF-β exposure.

*dpp* and *daw*, two homologs of TGF-β/MBP superfamily, are expressed in the pigment glia but not in photoreceptors (fig. S7A) (*49*). A reduction of the expression of *dpp* in pigment glia leads to an increase in GlcCer in neurons, consistent with the data observed with cultured neurons. *daw* may also play a role in stimulating GlcCer secretion, and the observed defects may be enhanced by the loss of *daw*. In summary, our data argue that the transfer for exosomes containing GlcCer is an evolutionarily conserved process that is based on a TGF-β/BMP signaling.

Increased numbers of EVs were reported in heads of *Gba1b* mutant flies (*62*), suggesting that increased levels of GlcCer lead to an increase in EVs. Exosomes secreted by neurons have been shown to facilitate the clearance of β-amyloid. Moreover, several studies suggest that there is a positive correlation between the levels of GlcCer and α-synuclein aggregation (*20*, *79*), and loss of *GBA1* enhances α-synuclein transmission between cells (*80*). In addition, neuron-to-glia transport of α-synuclein has been reported, but the precise mechanisms are yet to be elucidated (*81*). In *Drosophila*, overexpression of α-synuclein in photoreceptor neurons causes increased numbers of MVBs (*82*). These data indicate that neuron-to-glia transport of α-synuclein may share the same exosomes as those that transport GlcCer, and given the elevated levels of GlcCer in glia, aggregation of α-synuclein in glia may be favored. It has been argued that glial cells play an important role in the progression of PD. α-Synuclein accumulates in astrocytes and has been associated with the recruitment of phagocytic microglia that engulf selected neurons in restricted brain regions possibly causing the clinical symptoms associated with PD (*83*).

### White promotes GlcCer transport to lysosomes in glia

*white* encodes a transmembrane ABCG transporter (*84*). Its cellular and molecular functions are not well characterized, although it is the most commonly used marker in fly research. *white* is expressed in *Drosophila* pigment glia and at much lower levels in photoreceptor neurons (*30*, *31*). White is required for the formation of pigment granules in pigment glia and photoreceptors. RNAi-mediated knockdown of *white* in pigment glia results in a severe loss of eye pigmentation (fig. S4). Loss of *white* also reduces life span by about 10%, affects climbing ability starting at day 30, and causes a progressive retinal degeneration (*51*).

A reduction of *white* expression using RNAi in glia alone leads to glial expansion and accumulation of GlcCer after 3 days of a D/L cycle. However, this phenotype is strongly exacerbated by the loss of *Gba1b*. These data suggest that *white* is required for the degradation of GlcCer in glia and that its loss synergizes with the loss of *Gba1b*, arguing that both proteins have separate functions that converge on GlcCer degradation. The White protein colocalizes with the MVB marker Hrs but not with the lysosomal marker CTSL, indicating that White is associated with membrane trafficking in the MVBs. Moreover, White proteins together with MVBs are relocated to lysosomes after GlcCer treatment (fig. S5B), suggesting that White modulates GlcCer trafficking to lysosome through a pathway that relies on MVBs. We propose that both *white* and *Gba1b* are required for the degradation of GlcCer and that in the absence of one or the other, some degradation occurs but that in the absence of both, no degradation of GlcCer occurs, leading to a highly toxic combination that will quickly affect neuronal function and strongly accelerate the phenotypes. This also raises the possibility that other ABCG transporters may participate in GlcCer degradation in other glial cells. In summary, we propose that active neurons produce GlcCer. Glial cells provide a TGF-β/BMP signal that stimulates GlcCer secretion of exosomes from neurons that are then endocytosed by glial cells for degradation by Gba1b and White.

## MATERIALS AND METHODS

### Reagents and resources

### Experimental model and subject details

#### Drosophila

Flies were raised on molasses-based food at 25°C in constant dark unless otherwise noted. The full list of genotypes of fly stocks used can be found in the Key Resources Table.

#### Mammalian cell culture

Daoy and MO3.13 cells were used in this study. Cells were maintained in Dulbecco’s modified Eagle’s medium with GlutaMAX medium supplemented with 10% FBS, 1× pyruvate, and 1× penicillin and streptomycin.

#### Insect cell culture

S2 cells were used in this study. Cells were maintained in Schneider’s *Drosophila* Medium supplemented with 10% FBS and 1× penicillin and streptomycin (Tables 1 to 5).

**Table 1.** Experimental models: Organisms/strains. BDSC, Bloomington Drosophila Stock Center; VDRC, Vienna Drosophila Resource Center; N/A, not applicable.

	**Source**	**Identifier**
y^1^ w*; TI{GFP[3xP3.cLa]=CRIMIC.TG4.0}Gba1b^CR00541-TG4.0^/ TM3, Sb^1^ Ser^1^	BDSC	FBti0199405
w*; P{w[+mC]=UAS-mCherry.NLS}3	BDSC	FBst0038424
*w^1118^; dGBA1b*^−/−^, we renamed it to *Gba1b*^*STOP*/*STOP*^	N/A	Gift from K. Kinghorn (*22*)
w*; P{w[+mC]=UAS-RedStinger}4, P{w[+mC]=UAS- FLP.D}JD1, P{w[+mC]=Ubi-p63E(FRT.STOP) Stinger}9F6/CyO	BDSC	G-TRACE, FBst0028280
*y^1^ v^1^; P{y[+t7.7] v[+t1.8]=TRiP.JF01355}attP2*	BDSC	*Luciferase* RNAi, FBti0130444
y^1^ sc* v^1^ sev^21^; P{y[+t7.7] v[+t1.8]=TRiP.HMS01893} attP40	BDSC	*Gba1b* RNAi, FBst0038977
*P{KK107189}VIE-260B*	VDRC	*Gba1b* RNAi, FBst0473085
y^1^ w*; P{w[+m*]=GAL4}54C	BDSC	*54C-Gal4*, FBti0100692
y^1^ w*; Pbac{UAS-GBA1}/SM6a	This study	N/A
w*; Pbac{UAS-GBA1^N370S^}/CyO, Gal80; TM6,Tb/+	N/A	Gift from L. Pallanck (*21*)
*w^1118^; Df(3R)BSC490/TM6C, Sb^1^ cu^1^*	BDSC	Deficiency line for *Gba1b*, FBab0045306
y^1^ sc* v^1^ sev^21^; P{y[+t7.7] v[+t1.8]=TRiP.HMC06408} attP40	BDSC	*GlcT* RNAi, FBti0185552
*w^1118^; P{GD2142}v44912*	VDRC	*GlcT* RNAi, FBst0465833
*y^1^ v^1^; P{y[+t7.7] v[+t1.8]=TRiP.HMS00017}attP2*	BDSC	*w* RNAi, FBti0140096
y^1^ sc* v^1^ sev^21^; P{y[+t7.7] v[+t1.8]=TRiP.HMC06329} attP40	BDSC	*Glaz* RNAi, FBti0185474
*P{KK107553}VIE-260B*	VDRC	*Nlaz* RNAi, FBst0473194
y^1^ w*; PBac{CH322-118C10}VK00037	This study	20-kb BAC genomic rescue fragment for *Gba1b*
*y^1^ v^1^; P{y[+t7.7] v[+t1.8]=TRiP.HMS01844}attP40*	BDSC	*CHMP2B* RNAi, FBti0149590
*y1 v1; P{y[+t7.7] v[+t1.8]=TRiP.HMS02004}attP40*	BDSC	*Mvb12* RNAi, FBti0149746
y^1^ sc* v^1^ sev^21^; P{y[+t7.7] v[+t1.8]=TRiP.HMS01747} attP40	BDSC	*lns* RNAi, FBti0149494
*UAS-dTrpA1*	N/A	Gift from P. Garrity (*46*)
w*; UAS-dpp-RNAi-1+2-homo/CyO;TM6b (2885)	N/A	*dpp* RNAi, gift from K. Wharton (*60*)
*y^1^ v^1^; P{y[+t7.7] v[+t1.8]=TRiP.JF02455}attP2*	BDSC	*dpp* RNAi, FBti0146826
*y^1^ v^1^; P{y[+t7.7] v[+t1.8]=TRiP.JF02794}attP2*	BDSC	*dpp* RNAi, FBti0141012

**Table 2.** Experimental models: Cell lines. ATCC, American Type Culture Collection.

	Source	Identifier
*Drosophila melanogaster*: Cell line S2: S2-DRSC	Laboratory of N. Perrimon	CVCL_Z232; FlyBase: FBtc0000181
MO3.13	Cedarlane Labs	cat#CLU301
Daoy	ATCC	cat#ATCC-HTB-186

**Table 3.** Antibodies.

	Source	Identifier
Rat anti-elav	DSHB	AB_528218
Mouse anti-Repo	DSHB	AB_528448
Rabbit anti-Glc-Cer	Glycobiotech	cat#RAS_0010
Phalloidin 488 nm	Thermo Fisher Scientific	AB_2315147
Mouse anti-ATP5α	Abcam	AN_301447
DAPI	Thermo Fisher Scientific	AB_2629482
Alexa 488–conjugated secondary antibodies	Jackson ImmunoResearch Labs	AB_2338059
Alexa Cy3–conjugated secondary antibodies	Jackson ImmunoResearch Labs	AB_2338013
Alexa Cy5–conjugated secondary antibodies	Bioss Inc.	AB_11117143
Rabbit anti-Rab5	Abcam	ab31261
Mouse anti-CTSL	R&D Systems	MAB22591
Mouse anti-Rab7	DSHB	AB_2722471
Guinea pig anti–FL-Hrs	Bellen laboratory	GP30
Mouse monoclonal anti-HA	Covance	AB_10064220
Rabbit anti-HA	Sigma-Aldrich	H6908
C6-NBD-Glucosylceramide	Cayman Chemical	23209
C6-Biotin-Glucosylceramide	Cayman Chemical	23208
Hoechst 33342	Invitrogen	H3570
Mouse anti-CD63	Invitrogen	Ts63

**Table 4.** Chemicals. EMS, Electron Microscopy Sciences; Embed 812, EMBED 812 RESIN; NMA, (methyl-5-norbornene-2,3-dicarboxylic anhydride); DDSA, dodecenyl succinic anhydride specially distilled; DMP-30, DMP30.

	Source	Identifier
VECTASHIELD	Vector Labs	cat#H-1000
RapiClear	SunJin Lab Co.	N/A
Pierce Protease Inhibitor Tablets, EDTA-free	Thermo Fisher Scientific	cat#88266
Schneider’s *Drosophila* medium	Thermo Fisher Scientific	cat#21720
Fetal bovine serum, heat- inactivated	Sigma-Aldrich	cat#F4135
Penicillin streptomycin	Thermo Fisher Scientific	cat#15070063
Cacodylic acid, trihydrate sodium 100 g	EMS	cat#12300
EM-grade glutaraldehyde, 25% Aq solution	EMS	cat#16221
Osmium tetroxide 4% Aq solution	EMS	cat#19191
Paraformaldehyde 16% Aq solution	EMS	cat#15711
Propylene oxide	EMS	cat#20411
Koptec 200 Proof 100% ethanol anhydrous	VWR	cat#89125-186
Embed-812	EMS	cat#14901
NMA	EMS	cat#19001
DDSA	EMS	cat#13711
DMP-30	EMS	cat#13600
Uranyl acetate	EMS	cat#RT22400
Lead nitrate	EMS	cat#RT17900-25
Western Lightning Plus-ECL	PerkinElmer	cat#NEL105001EA
Recombinant human TGF-beta 3 protein	R&D Systems	243-B3

**Table 5.** Primers and recombinant DNA and siRNA.

	Source	Identifier
GTGCTCCCGACTGGCAGTTGC	This study	*Gba1b* PP1 forward primer
ACAGGCTTTGGGGAATGTTGG	This study	*Gba1b* PP1 reverse primer
GTACACTTCATGAGCATGGGCTG	This study	*Gba1b* PP2 forward primer
TGCAGGACTCCGTGTTCAATAGC	This study	*Gba1b* PP2 reverse primer
Plasmid: pUASTattb_white-HA	This study	N/A
Plasmid: pUASTattb_GBA1	This study	N/A
Glucosylceramidase beta siRNA #1	Thermo Fisher Scientific	s501314
Glucosylceramidase beta siRNA #2	Horizon Inspired Cell Solutions	J-006366-07-0005

### Method details

#### Life span

All flies of genotypes indicated in Fig. 1B were collected at eclosion and divided into multiple vials (20 flies per vial). They were raised at 25°C in a 12-hour dark/12-hour light cycle. All flies were transferred to fresh vials, and the number of dead flies was counted every 3 days. Survival rates were quantified from the total population.

#### Immunostaining

For immunostaining of fly retina, heads were dissected in cold 1× phosphate-buffered saline (PBS) and fixed in 3.7% formaldehyde diluted in PBS overnight at 4°C. On the second day, the retinae were dissected in cold PBS and fixed for an additional 30 min in 3.7% formaldehyde diluted in 1× PBS. Subsequently, the samples were washed with 0.1% Triton X-100 diluted in 1× PBS (0.1% PBST afterward). Washed samples were blocked in 5% natural goat serum (NGS) diluted in 0.1% PBST (5% NGST afterward) for 1 hour at room temperature (RT). After blocking, the samples were incubated overnight at 4°C with the following primary antibodies: rabbit anti-GlcCer (1:250; RAS_0010, Glycobiotech) and mouse anti-CTSL (1:1000; MAB22591, R&D Systems). On the third day, the samples were washed with 0.1% PBST, followed by incubation with Alexa 488–, Cy3-, or Cy5-conjugated secondary antibodies (111-545-144, 111-585-003, and 111-175-144, Jackson ImmunoResearch Labs and bs-2673R-Cy5.5, Bioss Inc.) and phalloidin 488 (AB_2315147, Thermo Fisher Scientific) for 1 hour at RT. After incubation with secondary antibodies, the samples were washed with 0.1% PBST. Last, the samples were mounted with VECTASHIELD (H-1000, Vector Laboratories) and kept at −20°C before imaging under a confocal microscope. All the confocal images were acquired with a Leica SP8 confocal microscope. All retinal confocal images were taken 5 μm below the surface. The fluorescent signals were separately quantified by outlining the neuronal or glial area, respectively. Confocal images were processed using Fuji (ImageJ) and Photoshop (Adobe).

For immunostaining of fly brains, larval and adult brains were dissected in cold 1× PBS and fixed in 4% paraformaldehyde diluted in 1× PBS overnight at 4°C. On the second day, the fixed brains were permeabilized with 2% Triton X-100 diluted in 1× PBS overnight at 4°C. The samples were blocked in 5% NGST under vacuum at RT for 2 hours. After blocking, the samples were incubated overnight at 4°C with the following primary antibodies: rat anti-elav [1:250; Rat-elav-7E8A10, DSHB (Developmental Studies Hybridoma Bank)] and mouse anti-Repo (1:50; Mouse-Repo-8D12, DSHB). Then, the samples were washed with 0.1% PBST, followed by incubation with Alexa 488–, Cy3-, or Cy5-conjugated secondary antibodies (111-545-144, 111-585-003, and 111-175-144, Jackson ImmunoResearch Labs and bs-2673R-Cy5.5, Bioss Inc.) for 1 hour at RT. Last, the samples were mounted with RapiClear (SunJin Lab Co.) and kept at −20°C before imaging under a confocal microscope. All the confocal images were acquired with a Leica SP8 confocal microscope. Confocal images were processed using Fuji (ImageJ) and Photoshop (Adobe).

For immunostaining of S2 cells, S2 cells were cultured on poly-d-lysine (PDL)–treated coverslips and fixed in 4% paraformaldehyde diluted in 1× PBS for 10 min at RT. Fixed cells were rinsed with 1× PBS followed by permeabilizing with 0.2% Triton X-100 diluted in 1× PBS for 10 min at RT. Subsequently, the cells were blocked with 1% bovine serum albumin diluted with 1× PBS for 1 hour at RT. After blocking, the samples were incubated overnight at 4°C with the following primary antibodies: mouse anti-Atp5α (1:250; Mouse-Repo-α5, DSHB), mouse anti-CTSL (1:1000; MAB22591, R&D Systems), rabbit anti-Rba5 (1:250; ab31261, Abcam), mouse anti-Rab7 (1:250; AB_2722471, DSHB), and guinea pig anti–FL-Hrs (1:250; GP30). Then, the samples were washed with 1× PBST, followed by incubation with Alexa 488–, Cy3-, or Cy5-conjugated secondary antibodies (111-545-144, 111-585-003, and 111-175-144, Jackson ImmunoResearch Labs and bs-2673R-Cy5.5, Bioss Inc.) for 1 hour at RT. Last, the samples were mounted with 4′,6-diamidino-2-phenylindole (DAPI) Fluoromount-G (SouthernBiotech) and kept at −20°C before imaging under a confocal microscope. All the confocal images were acquired with a Leica SP8 confocal microscope. Confocal images were processed using Fuji (ImageJ) and Photoshop (Adobe).

#### ERG recording

ERG recordings were performed by following the protocol documented in (*85*). Flies, as indicated in the manuscript, were glued on glass slides. Two electrodes were filled with 0.1 M NaCl. One of them was probed into the fly thorax, and the other one was placed on the fly eye. A 1-s pulse of light stimulation interval was shined. The ERG traces were recorded and analyzed by LabChart 8 Reader.

#### Transmission electron microscopy

The ultrastructure of the *Drosophila* visual system was processed following standard electron microscopy procedures as documented in (*11*). Adult heads were dissected and fixed in fixative [2% paraformaldehyde, 2.5% glutaraldehyde, 0.1 M sodium cacodylate, and 0.005% CaCl_2_ (pH 7.2)] at 4°C overnight and then postfixed in 1% OsO_4_. The fixed samples were dehydrated in ethanol and propylene oxide and then embedded in Embed-812 resin (Electron Microscopy Sciences) under vacuum. Photoreceptors were then sectioned and stained in 1% uranyl acetate and saturated lead nitrate. TEM images of photoreceptor sections were taken using a JEOL JEM 1010 transmission electron microscope at 80 kV with an AMT XR-16 mid-mount 16-megapixel digital camera.

#### Coculture assay

Cells were split and cultured on PDL-coated coverslips in 24-well plates at a density of 5 × 10^4^ the day before the experiment. On the day of the assay, cells were washed with cold Hanks’ balanced salt solution (HBSS; Gibco). The cells were then incubated with 4 μM C6-NBD-GlcCer (Cayman) at 4°C for 30 min. C6-NBD-GlcCer is dissolved in cold HBSS by vortexing to create micelles. At 4°C, the plasma membrane of cells is labeled with C6-NBD-GlcCer. After labeling, the cells are rinsed with warm HBSS (37°C) to remove unlabeled C6-NBD-GlcCer. Subsequently, the cells were transferred in a cell culture incubator (37°C and 5% CO_2_) to avoid light for 30 min and allow C6-NBD-GlcCer to be taken up by cells. After C6-NBD-GlcCer labeling, a paraffin ring was placed on top of the labeled cells followed by placing nonlabeled cells on top of the paraffin ring. The two coverslips with labeled cells (beneath the paraffin ring) and nonlabeled cells (above the paraffin ring) were face-to-face with each other and cocultured in the warm (37°C) ACSF [125 mM NaCl, 5 mM KCl, 2 mM MgSO_4_, 24 mM NaHCO_3_, 1.25 mM NaH_2_PO_4_, 2 mM CaCl_2_, and 10 mM glucose (pH 7.4), filtered with a 0.22-nm filter] in a cell culture incubator (37°C and 5% CO_2_) to avoid light for 30 min. After coculture, cells were mounted in warm HBSS with Hoechst, and live images were taken immediately with a Leica SP8 confocal microscope.

#### Conditional ACSF media incubation assay

Cells were cultured in 15-cm dishes and reached confluency in normal cell culture media as mentioned above in the “Mammalian cell culture” section. Cells were then washed with warm ACSF twice and incubated with ACSF in a cell culture incubator (37°C and 5% CO_2_) overnight the day before the experiment. On the day of the assay, conditional ACSF media were harvested and filtered with 0.45-nm filters to remove cellular debris. Cells were labeled with C6-NBD-GlcCer as mentioned above. The labeled cells were then incubated with conditional ACSF media, as documented in Results, in a cell culture incubator (37°C and 5% CO_2_) to avoid light for 30 min. After conditional ACSF media incubation, cells were mounted in warm HBSS with Hoechst, and live images were taken immediately with a Leica SP8 confocal microscope.

#### FBS (10%) and TGF-β treatment assay

Cells were labeled with C6-NBD-GlcCer as mentioned above. Subsequently, labeled cells were incubated with 10% FBS or different concentrations of TGF-β diluted in warm ACSF in a cell culture incubator (37°C and 5% CO_2_) to avoid light for 30 min. After treatment, cells were mounted in warm HBSS with Hoechst, and live images were taken immediately with a Leica SP8 confocal microscope.

#### Exosome isolation assay

Cells were cultured on 10-cm plates to confluency (five plates per experimental condition). They were then labeled with C6-NBD-GlcCer. Subsequently, labeled cells were incubated with or without 50 nM TGF-β diluted in warm ACSF in a cell culture incubator (37°C and 5% CO_2_) to avoid light for 1 hour. After treatment, ACSF media were harvested, and total exosomes from the media were purified by using Total Exosome Isolation Reagent (Invitrogen). Then, CD63-positive exosomes were isolated from total exosomes by using Exosome-Human CD63 Isolation/Detection Reagent (Invitrogen).

#### Lipid extraction

Lipids were extracted as described previously (*86*). For hexosylceramide analysis from fly heads, deuterated GlcCer (d18:1/16:0 d3-GlcCer) was added to 1000 fly heads as an internal standard with 500 μl of 1-butanol:methanol (1:1, v/v). For multiplexed ceramides, hexosylceramides, and hexosylsphingosine analysis from Daoy cells and media, deuterated GlcCer (d18:1/16:0 d3-GlcCer), ceramide (d18:1 d7/18:0-Cer), and lyso globotriaosylsphingosine (lyso Gb3 d7) were added. Homogenates were sonicated for 60 min at RT and centrifuged at 13,000*g* for 10 min. The supernatant was dried using a vacuum centrifuge and reconstituted in chloroform:methanol (1:4, v/v) and centrifuged at 13,000*g* for 5 min to remove any insoluble salts. The supernatant was collected and injected for liquid chromatography–tandem mass spectrometry (LC-MS/MS) for lipid analysis.

#### Targeted analysis of lipids using LC-MS/MS

Vanquish Horizon UHPLC (Thermo Fisher Scientific, Waltham, MA) was coupled to an Orbitrap Fusion Tribrid ID-X mass spectrometer (Thermo Fisher Scientific, Waltham, MA) for analysis of lipids. Two targeted methods of lipids were developed using Hypersil Gold Vanquish C_18_ UHPLC column (2.1 mm × 15 cm, 1.9 μm), one for hexosylceramides and another for ceramides, hexosylceramides, sphingosine, and hexosylsphingosine. For both methods, a binary gradient of mobile phase A (water:acetonitrile, 6:4, v/v) and mobile phase B (isopropanol:methanol:acetonitrile, 8:1:1, v/v/v) with 0.1% formic acid and 10 mM ammonium formate at the flow rate of 300 μl/min was applied to separate lipids. Full-scan MS spectra at a resolution of 60,000 [mass/charge ratio (*m/z*) 200] and MS/MS spectra at a resolution of 15,000 (*m/z* 200) in scheduled parallel reaction monitoring mode were obtained with a column temperature of 50°C and a spray voltage of 3.5 kV in positive ion mode. Because of differences in target lipids between the two methods, slightly different parameters of LC-MS/MS including LC gradient, full-scan MS range, and collision energy in higher-energy collisional dissociation were applied.

For targeted analysis of hexosylceramides, mobile phase B was increased from 50 to 95% over 7 min, 95% for 6 min, 50% over 0.1 min, and 50% B for 4 min. A primary MS scan of 400 to 850 *m/z* was carried out to cover the range of hexosylceramides, and higher-energy collisional dissociation was set at 40%. For targeted analysis of multiplexed ceramides, hexosylceramides, and hexosylsphingosine, mobile phase B was increased from 20 to 60% over 2 min, 75% over 2.5 min, 85% over 1.5 min, 95% over 1 min, maintained at 95% for 6 min, 20% for 0.1 min, and equilibrated for 4 min. A full-scan MS of 250 to 900 *m/z* was acquired, and collision energy was set at 35% for ceramides and hexosylsphingosine and 40% for dihydroceramides and hexosylceramides.

Peak areas of lipids were calculated using Skyline (MacCoss Lab Software), normalized to the peak area of the deuterated internal standard, and corrected by the protein amount measured from the bicinchoninic acid assay. The total level of lipid for each class was calculated by adding the normalized peak areas of individual species.

### Quantification and statistical analysis

All datasets were organized and analyzed in Microsoft Excel (version 2019) and GraphPad Prism (version 9.2.0) using two-tailed Student’s *t* test or one-way analysis of variance (ANOVA). For fly experiments, sample sizes are stated in the figure legends. All crosses were performed at least twice. For cell experiments, all studies were conducted for a minimum of three biological replicates. Error bars are shown as SEM. The criteria for significance are as follows: ns (not significant), *P* > 0.05; *P < 0.05; **P < 0.01, ***P < 0.001, and ****P < 0.0001.

## Supplementary Materials

### This PDF file includes:

Figs. S1 to S7 References

## Notes

View/request a protocol for this paper from *Bio-protocol*.
