# Supplementary material for: Neuronal activity induces glucosylceramide that is secreted via exosomes for lysosomal degradation in glia
Source: Sci Adv. 2022 Jul 13;8(28):eabn3326. doi: 10.1126/sciadv.abn3326 (PMC9278864; doi:10.1126/sciadv.abn3326)
Supplement: Supplementary file 1 — Figs. S1 to S7 References [file sciadv.abn3326_sm.pdf]

Science Advances
AAAS

Supplementary Materials for

Neuronal activity induces glucosylceramide that is secreted via exosomes for lysosomal degradation in glia

Liping Wang *et al.*

Corresponding author: Hugo J. Bellen, hbellen@bcm.edu

Sci. Adv. **8**, eabn3326 (2022)
DOI: 10.1126/sciadv.abn3326

This PDF file includes:

Figs. S1 to S7
References

Figure S1

A. $y^1 w^*; Gba1b^{T2A\text{-}Gal4}$ **is a null allele**

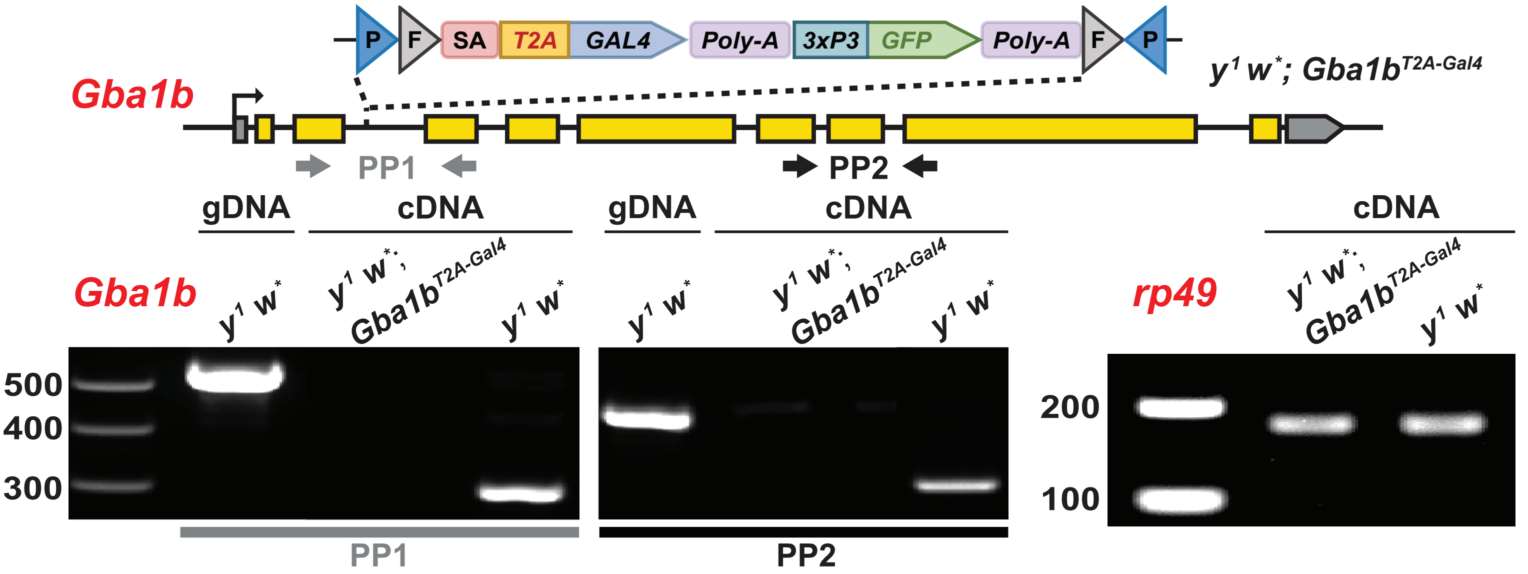

B. Loss of *Gba1b* leads to increased GlcCer levels in fly brain

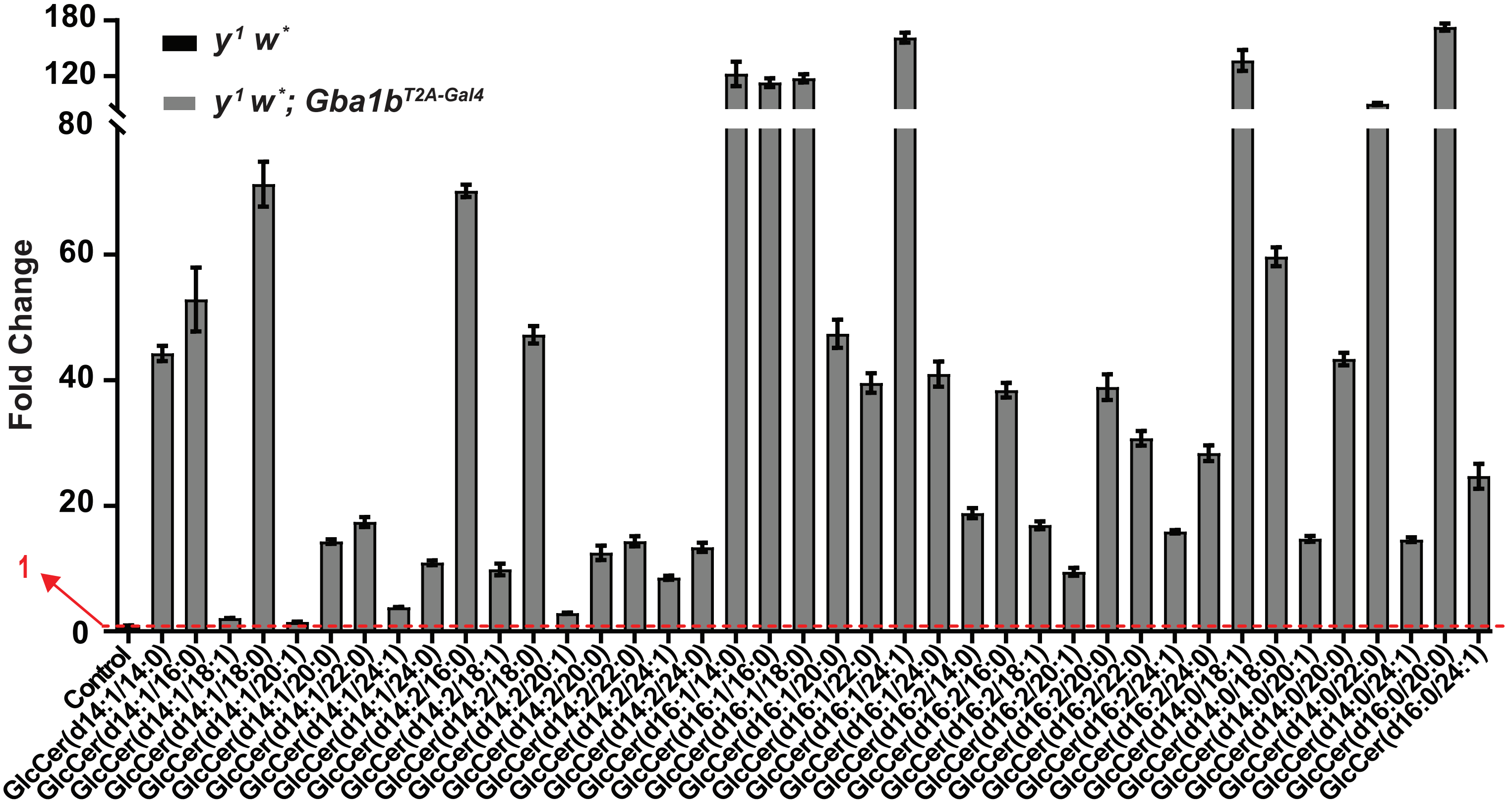

C. *G-Trace* is used to determine both real-time and lineage expression of gene of interst

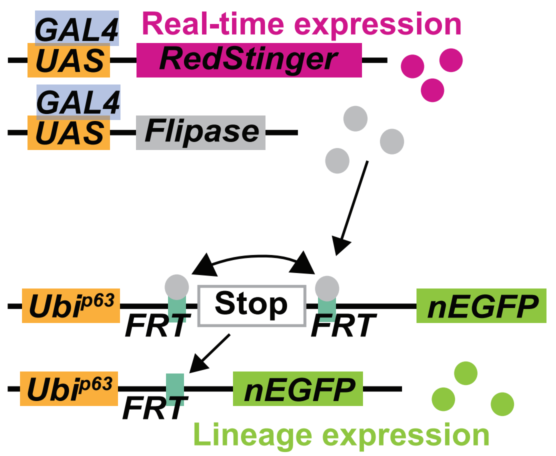

D. *Gba1b* is not expressed in neurons during development

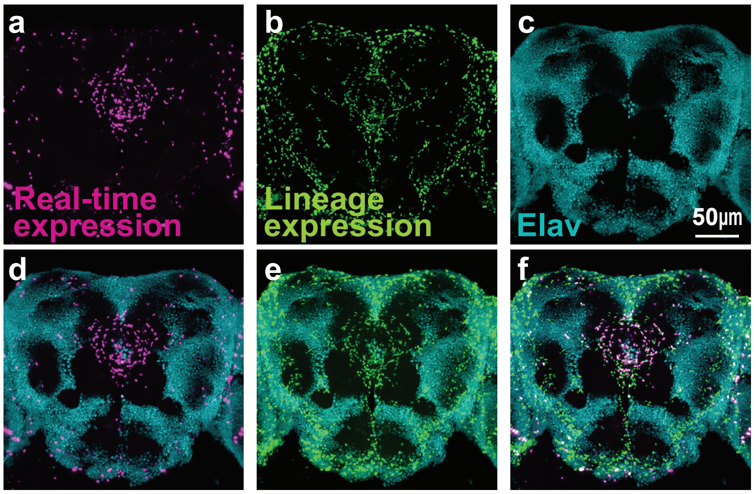

Figure S1 to Figure 1. ***y¹ w*; Gba1b*** $^{T2A\text{-}Gal4}$ **is a null allele**

(**A**) RT-PCR results of the indicated genotypes. Two primer pairs are used: PP1 (gray) and PP2 (black). No product is amplified using PP1 in *y¹ w*; Gba1b*$^{T2A\text{-}Gal4/T2A\text{-}Gal4}$ (*y¹ w*; Gba1b*$^{T2A\text{-}Gal4}$ for short afterwards), indicating that the CRIMIC cassette is inserted in this region. Moreover, no RT-PCR product is detected using PP2 in *y¹ w*; Gba1b*$^{T2A\text{-}Gal4}$, suggesting that transcription of *Gba1b* is arrested by the *PolyA* sequence in the CRIMIC cassette. Hence, *y¹ w*; Gba1b*$^{T2A\text{-}Gal4}$ is a severe loss of function or null allele. Flies that are tested in this experiment are w^-. (**B**) Lipidomics results of the indicated genotypes. lipidomic assays are performed to compare levels of GlcCer between *y¹ w** and *y¹ w*; Gba1b*$^{T2A\text{-}Gal4}$ fly heads. The results show that on average there is an approximate 16-fold increased level of total GlcCer in *y¹ w*; Gba1b*$^{T2A\text{-}Gal4}$ mutants than *y¹ w** controls. (N = 3). Flies that are tested in this experiment are w^-. (**C**) Schematic of G-TRACE technology (*38*). GAL4 protein produced in *y¹ w*; Gba1b*$^{T2A\text{-}Gal4}$ activate *UAS-RedStinger* and *UAS-Flipase*. RFP represent cells expressing *Gba1b* in real-time. Cells expressing the Flipase recombinase excise the Stop cassette flanked by two *FRT* sites leading to the expression of a nuclear EGFP (*nEGFP*) which is inherited in all daughter cells and hence reports historical expression pattern. (**D**) Magenta and green signals represent real-time and historical expression of *Gba1b*, respectively. The Elav (Canyon) antibody labels the neuronal nuclei. Again, *Gba1b* is only expressed in glia but not in neurons. (N = 3).

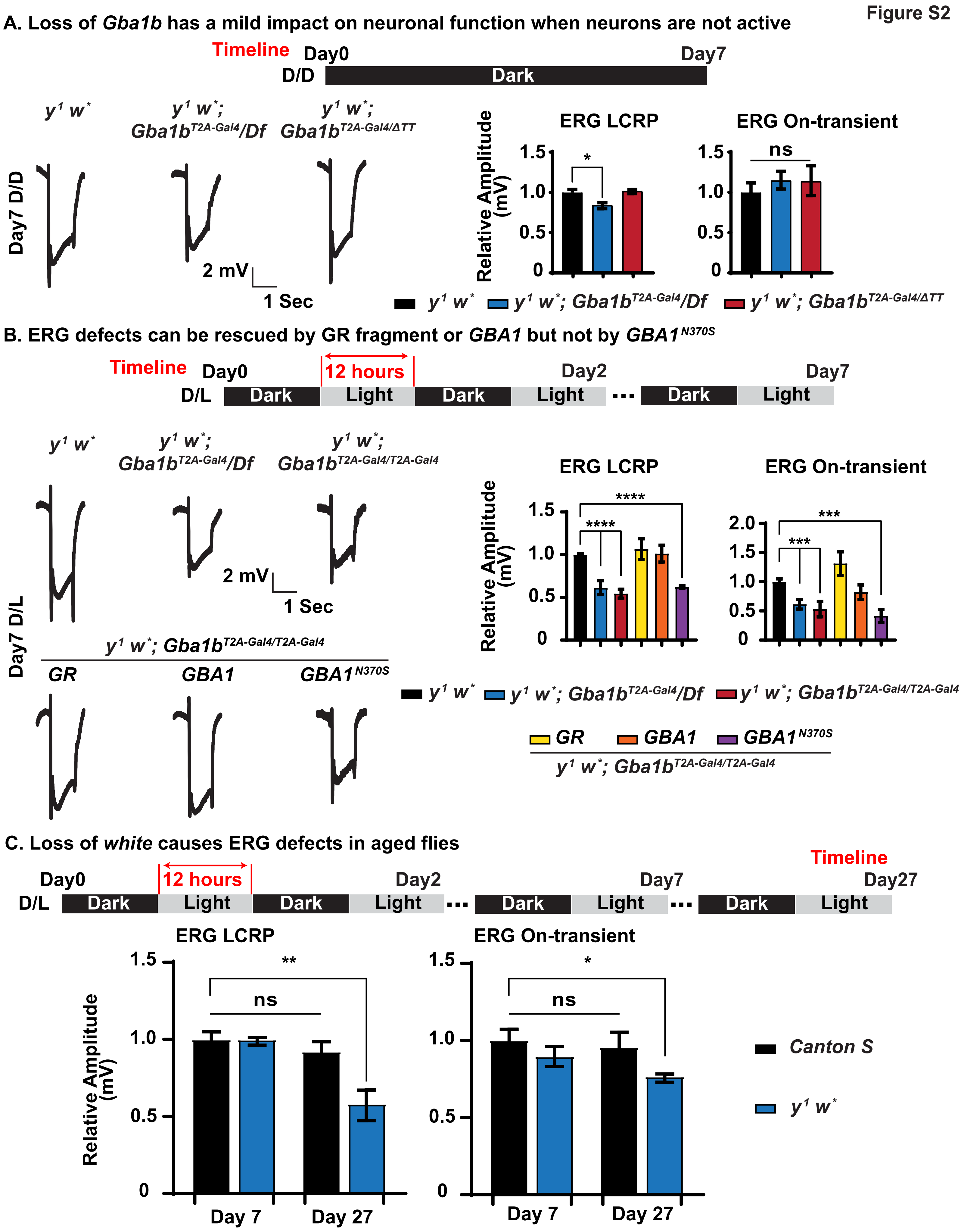
Figure S2
A. Loss of Gba1b has a mild impact on neuronal function when neurons are not active
Timeline
Day0
Day7
D/D
Dark
y1 w*
y1 w*; Gba1bT2A-Gal4/Df
y1 w*; Gba1bT2A-Gal4/ΔTT
Day7 D/D
2 mV
1 Sec
ERG LCRP
ERG On-transient
Relative Amplitude (mV)
1.5
1.0
0.5
0
*
ns
y1 w*
y1 w*; Gba1bT2A-Gal4/Df
y1 w*; Gba1bT2A-Gal4/ΔTT
B. ERG defects can be rescued by GR fragment or GBA1 but not by GBA1N370S
Timeline
Day0
12 hours
Day2
Day7
D/L
Dark
Light
Dark
Light
Dark
Light
y1 w*
y1 w*; Gba1bT2A-Gal4/Df
y1 w*; Gba1bT2A-Gal4/T2A-Gal4
2 mV
1 Sec
Day7 D/L
y1 w*; Gba1bT2A-Gal4/T2A-Gal4
GR
GBA1
GBA1N370S
ERG LCRP
ERG On-transient
Relative Amplitude (mV)
****
****
***
***
2.0
1.5
1.0
0.5
0
y1 w*
y1 w*; Gba1bT2A-Gal4/Df
y1 w*; Gba1bT2A-Gal4/T2A-Gal4
GR
GBA1
GBA1N370S
y1 w*; Gba1bT2A-Gal4/T2A-Gal4
C. Loss of white causes ERG defects in aged flies
Timeline
Day0
12 hours
Day2
Day7
Day27
D/L
Dark
Light
Dark
Light
Dark
Light
Dark
Light
ERG LCRP
ERG On-transient
Relative Amplitude (mV)
1.5
1.0
0.5
0
**
ns
*
ns
Day 7
Day 27
Canton S
y1 w*

Figure S2 to Figure 2. Neuronal defects caused by loss of *Gba1b* is light/activity dependent

(**A**) Light exposure is required to induce reduced LCRP and On-transient. ERG recordings of 10-day-old flies that were kept in constant darkness of the indicated genotypes. *y^1 w*; $Gba1b^{T2A\text{-}Gal4}$/Df* have slightly reduced LCRPs and wild type ERG On-transient. The ERG LCRP and on-transient amplitudes are quantified on the right. Error bars represent SEM (n≥ 6); *p < 0.05. Flies that are tested in this experiment are w^-. (**B**) Human *GBA1* rescue loss of *Gba1b* defects. ERG recordings of flies of the indicated genotypes after 7-days D/L cycles. Null allelic combinations (*y^1 w*; $Gba1b^{T2A\text{-}Gal4}$/Df* and *y^1 w*; $Gba1b^{T2A\text{-}Gal4/T2A\text{-}Gal}$*) show reduced ERG LCRP and ERG on-transient amplitudes. These defects can be rescued by introduction of genomic fragment containing *Gba1b* or expression of Human *GBA1* (these flies are red eyed because of w^+ transgene). The ERG LCRP and ERG on-transient amplitudes are quantified on the right. Error bars represent SEM (n ≥ 6); ***p < 0.001, ***p < 0.0001. (**C**) Loss of *white* causes reduced ERG LCRP and on-transient amplitudes in aged flies. y^1 w^* flies show similar ERG LCRP and on-transient amplitudes with *Canton S* flies at Day 7, whereas they exhibit reduced ERG amplitudes at Day 27 compared with *Canton S* flies. Error bars represent SEM (n ≥ 6); *p < 0.05, **p < 0.01.

Figure S3

A. Loss of *Gba1b* leads to vacuolized glia

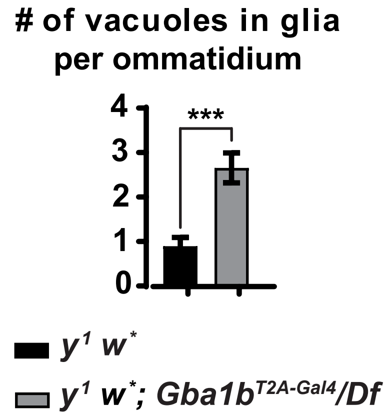

B. Loss of *Gba1b* leads to glial detachment from neurons

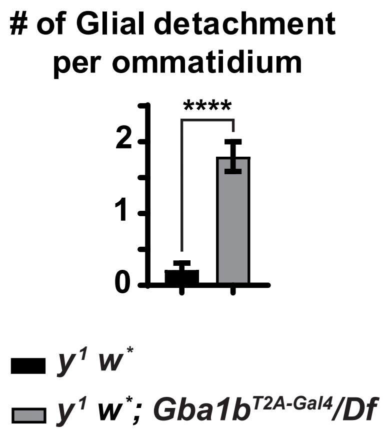

C. Loss of *Gba1b* leads to increased number of lysosomes in glia

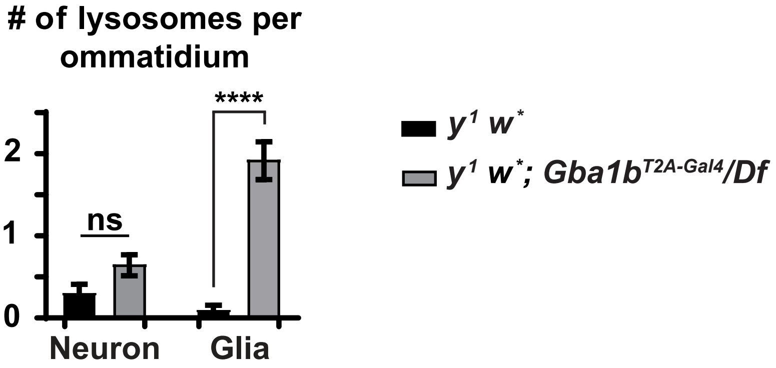

Figure S3 to Figure 3. Loss of *Gba1b* impairs glial morphology

Quantifications of the number of vacuoles (**A**), number of glial detachments (**B**), and number of lysosomes per ommatidium (**C**) in Figure 3A. Error bars represent SEM ($n = 3$); $^{***}p < 0.001$, $^{****}p < 0.0001$.

Figure S4

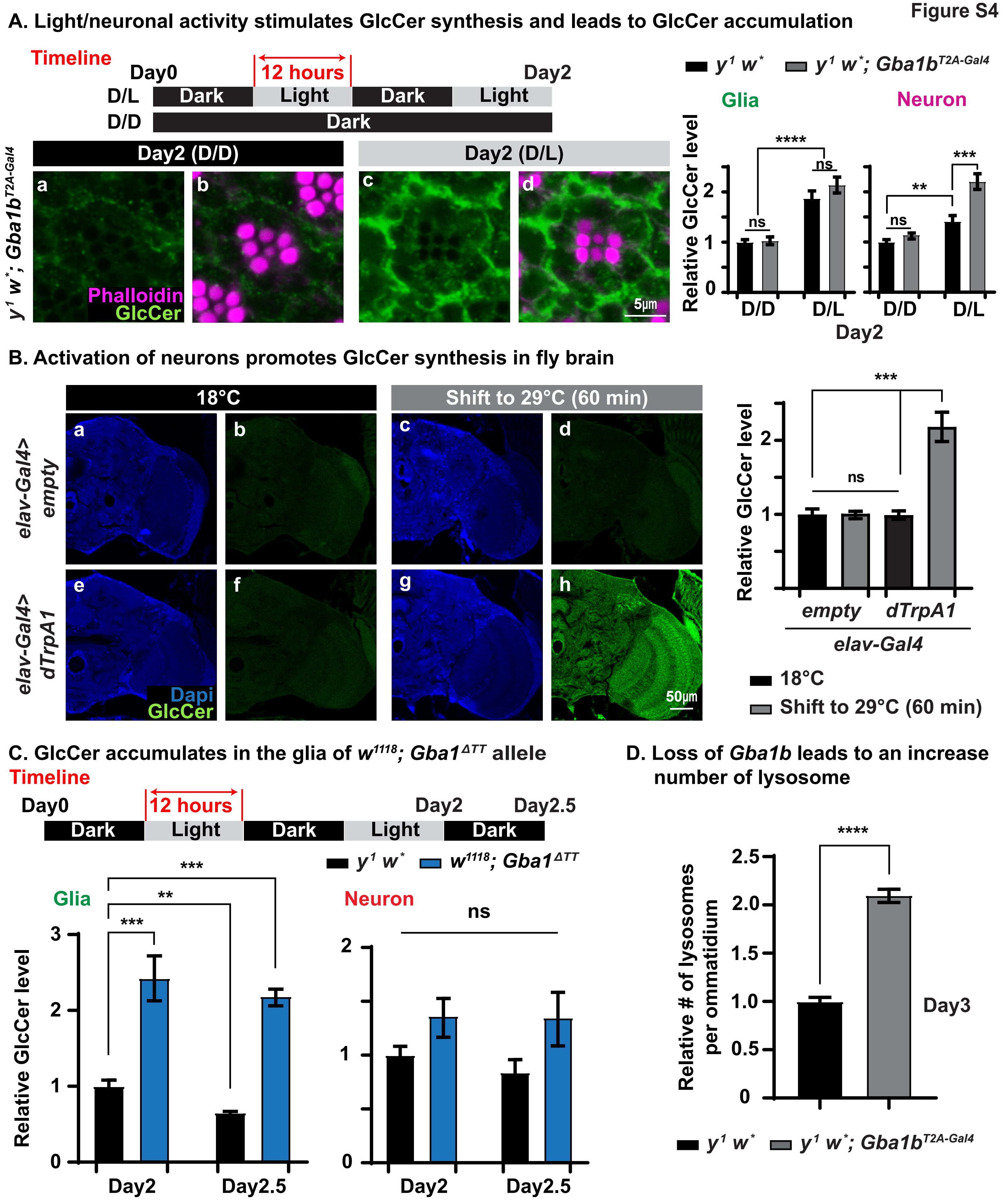

A. Light/neuronal activity stimulates GlcCer synthesis and leads to GlcCer accumulation
Timeline
Day0
12 hours
Day2
D/L
Dark
Light
Dark
Light
D/D
Dark
Day2 (D/D)
Day2 (D/L)
y1 w*; Gba1bT2A-Gal4
a
b
c
d
Phalloidin
GlcCer
5μm
y1 w*
y1 w*; Gba1bT2A-Gal4
Glia
Neuron
Relative GlcCer level
****
ns
ns
***
**
ns
D/D
D/L
D/D
D/L
Day2
B. Activation of neurons promotes GlcCer synthesis in fly brain
18°C
Shift to 29°C (60 min)
elav-Gal4> empty
elav-Gal4> dTrpA1
a
b
c
d
e
f
g
h
Dapi
GlcCer
50μm
***
ns
Relative GlcCer level
empty
dTrpA1
elav-Gal4
18°C
Shift to 29°C (60 min)
C. GlcCer accumulates in the glia of w1118; Gba1ΔTT allele
Timeline
Day0
12 hours
Day2
Day2.5
Dark
Light
Dark
Light
Dark
y1 w*
w1118; Gba1ΔTT
Glia
Neuron
***
**
***
ns
Relative GlcCer level
Day2
Day2.5
Day2
Day2.5
D. Loss of Gba1b leads to an increase number of lysosome
****
Relative # of lysosomes per ommatidium
Day3
y1 w*
y1 w*; Gba1bT2A-Gal4

Figure S4 to Figure 4. Neuronal activity promotes GlcCer production in neurons

(**A**) (a-b) Flies kept in the dark show very little GlcCer in ommatidia of *y*^{1} *w*; Gba1b*$^{T2A\text{-}Gal4}$ (c-d) After 12 hours light exposure GlcCer is synthesized and accumulates in neurons and glia of and *y*^{1} *w*; Gba1b*$^{T2A\text{-}Gal4}$ flies. ($n \geq 9$) Quantifications of relative GlcCer level in Figure 4A and S4A are shown in the lower graphs. Error bars represent SEM ($n \geq 9$); **$p < 0.01$, ***$p < 0.001$. (**B**) *trpA1* is overexpressed in fly neurons driven by e*lav-GAL4* (*46*). *trpA1* encodes a thermosensitive transient receptor potential channel (TRP channel) that functions in sensory neurons. It is a cation channel that is not permeable to Ca^{2+} and Na^{+} ions at 18 °C but and is highly permeable to these ions at 29 °C (*45*). Transgenic flies (e*lav-GAL4 >dTrpA1*) and negative control flies (e*lav-GAL4 >empty*) were raised at 18°C till there days after eclosion. They were then exposed to 29 °C for 60 min to promote neuronal activity. Immunostaining is performed on paraffin brain sections. (a-d) No significant differences in the GlcCer levels were observed in e*lav-GAL4 >empty* fly brain sections. (e-h) However, a significant increase in GlcCer levels were observed in many areas of the e*lav-GAL4 >dTrpA1* fly brains after the temperature shift, indicating that neuronal activity promotes GlcCer synthesis in the CNS. Quantifications of relative GlcCer level are shown in right. Error bars represent SEM ($n = 6$); ***$p < 0.001$. (**C**) Given the presence of 3XP3-GFP in the *y*^{1} *w*; Gba1b*$^{T2A\text{-}Gal4}$ allele (Figure 1A), we also assessed the levels of GlcCer in the *w*^{1118}; *GBA1*$^{\Delta TT}$ flies. Increased GlcCer levels are observed in the glia of *w*^{1118}; *GBA1*$^{\Delta TT}$ fly retina that lack 3XP3-GFP after 2 days of D/L cycle. Quantifications of relative GlcCer level is shown. Error bars represent SEM ($n \geq 9$); ***$p < 0.001$. (**D**) Number of lysosomes in Figure 4C were quantified. Error bars represent SEM ($n \geq 9$), ****$p < 0.0001$.

Figure S5

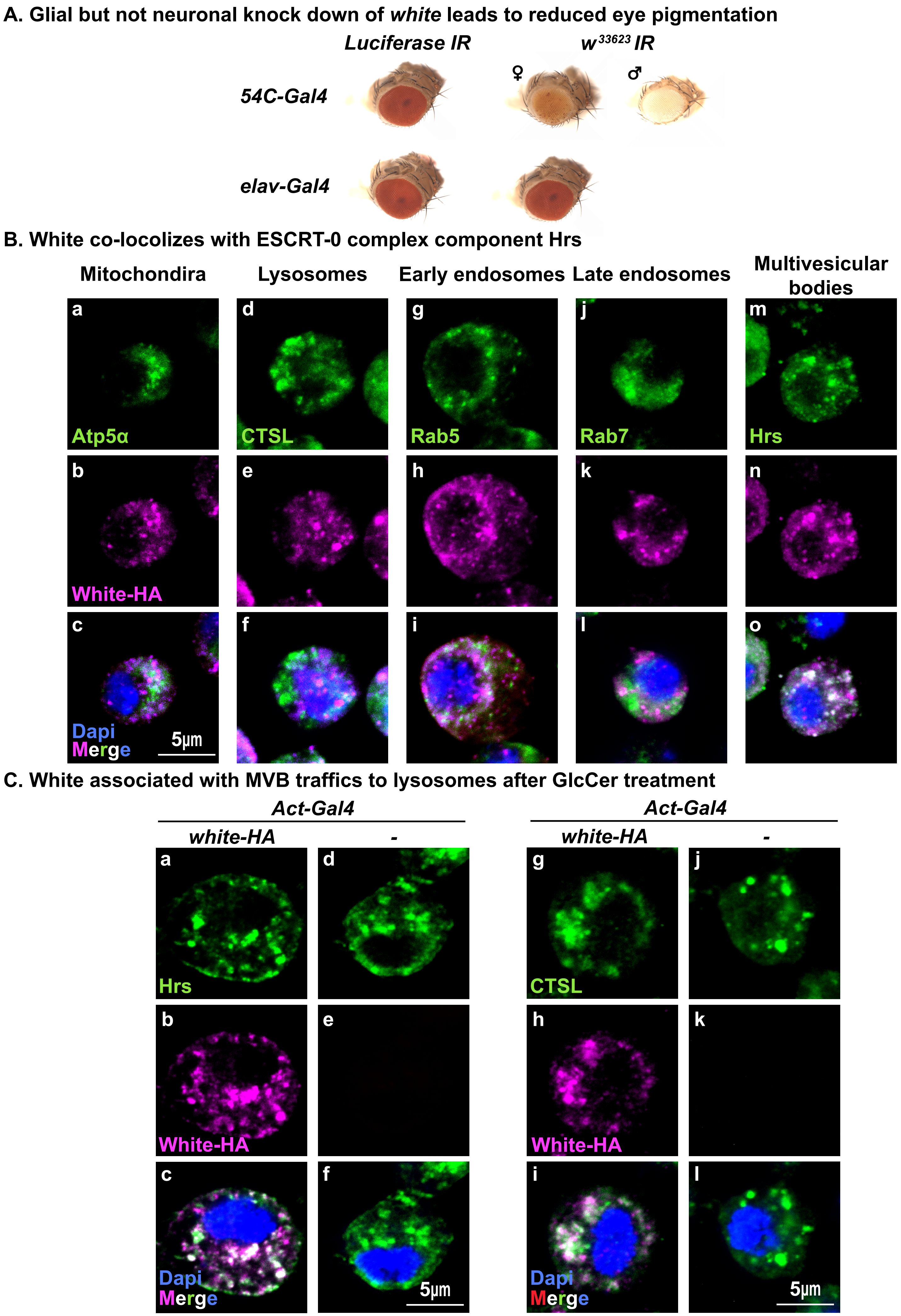

A. Glial but not neuronal knock down of white leads to reduced eye pigmentation
Luciferase IR
w33623 IR
♀
♂
54C-Gal4
elav-Gal4
B. White co-locolizes with ESCRT-0 complex component Hrs
Mitochondira
Lysosomes
Early endosomes
Late endosomes
Multivesicular bodies
a
d
g
j
m
Atp5α
CTSL
Rab5
Rab7
Hrs
b
e
h
k
n
White-HA
c
f
i
l
o
Dapi
Merge
5μm
C. White associated with MVB traffics to lysosomes after GlcCer treatment
Act-Gal4
white-HA
-
Act-Gal4
white-HA
-
a
d
g
j
Hrs
CTSL
b
e
h
k
White-HA
White-HA
c
f
i
l
Dapi
Merge
5μm
Dapi
Merge
5μm

Figure S5 to Figure 5. White is associated with MBVs and plays roles in glia of *Drosophila*

(**A**) Images of fly eye of the indicated genotypes. The *white* was knocked down using RNAi construct in neurons driven by *54C-Gal4* (upper panel) and in glia driven by *elav-Gal4* (lower panel), respectively. (**B**) The White protein colocalizes with Multivesicular Body (MVB) marker Hrs. S2 cells were transfected with *Act-Gal4* and *white-HA*. Immunofluorescent staining of transfected S2 cells. white-HA (magenta) indicates the subcellular localization of the White protein. Five cellular markers (green) were tested. White-HA colocalizes with the multivesicular body marker Hrs. (**C**) GlcCer promotes White to localize to lysosomes. S2 cells were transfected with *Act-Gal4* and *white-HA* or with *Act-Gal4* only. They were then treated with 4μM Biotin-GlcCer for 30 min. Immunofluorescent staining of treated S2 cells. White-HA (magenta) indicates the subcellular localization of the White protein. (a-f) White is associated with multivesicular body marker Hrs. (g-l) White associated with Hrs traffics to lysosomes after Biotin-GlcCer treatment.

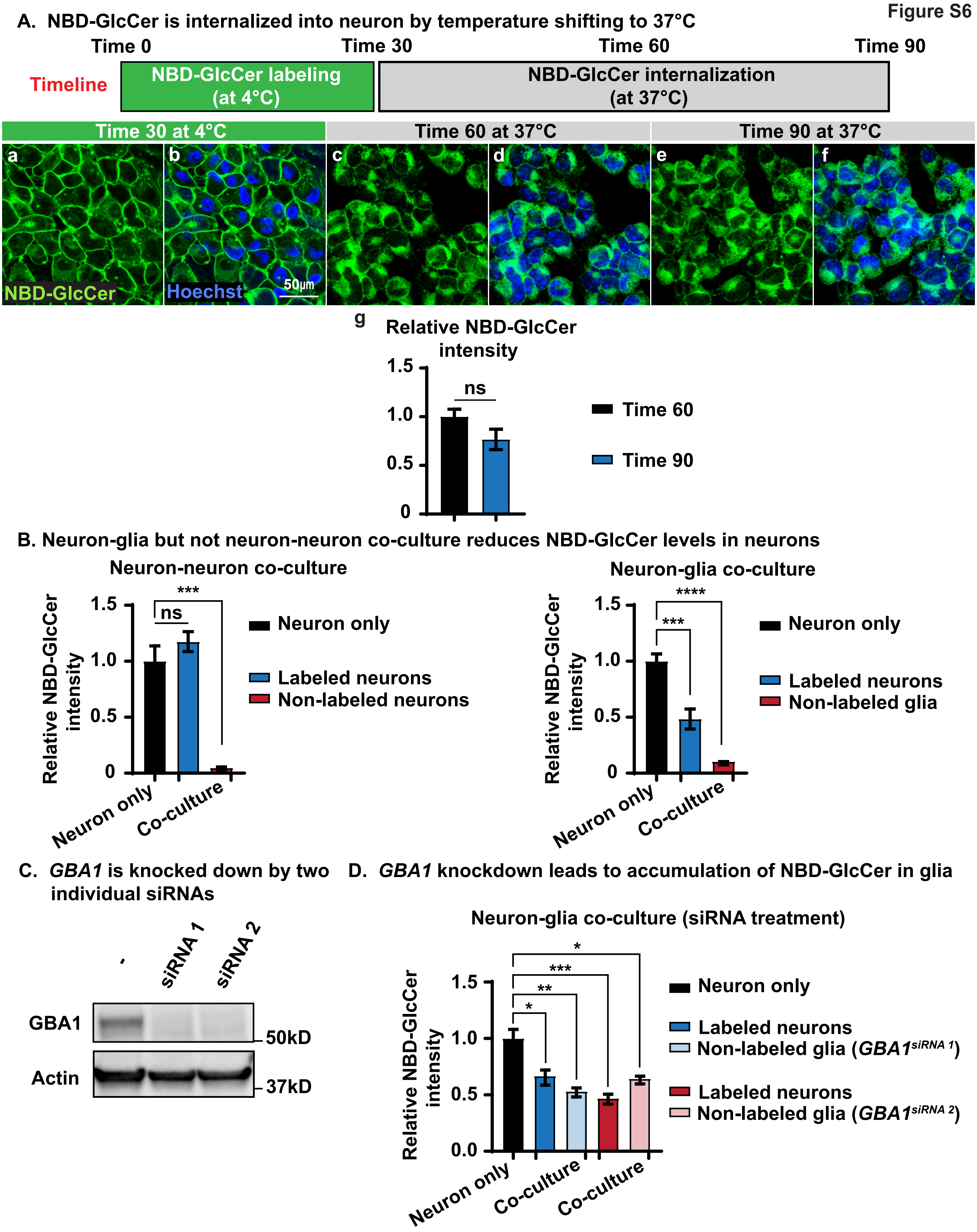
Figure S6
A. NBD-GlcCer is internalized into neuron by temperature shifting to 37°C
Time 0
Time 30
Time 60
Time 90
Timeline
NBD-GlcCer labeling (at 4°C)
NBD-GlcCer internalization (at 37°C)
Time 30 at 4°C
Time 60 at 37°C
Time 90 at 37°C
a
b
c
d
e
f
NBD-GlcCer
Hoechst
50μm
g
Relative NBD-GlcCer intensity
ns
Time 60
Time 90
B. Neuron-glia but not neuron-neuron co-culture reduces NBD-GlcCer levels in neurons
Neuron-neuron co-culture
Relative NBD-GlcCer intensity
ns
***
Neuron only
Labeled neurons
Non-labeled neurons
Neuron only
Co-culture
Neuron-glia co-culture
****
***
Neuron only
Labeled neurons
Non-labeled glia
C. GBA1 is knocked down by two individual siRNAs
-
siRNA 1
siRNA 2
GBA1
Actin
50kD
37kD
D. GBA1 knockdown leads to accumulation of NBD-GlcCer in glia
Neuron-glia co-culture (siRNA treatment)
*
***
**
*
Neuron only
Labeled neurons
Non-labeled glia (GBA1siRNA 1)
Labeled neurons
Non-labeled glia (GBA1siRNA 2)
Relative NBD-GlcCer intensity
Neuron only
Co-culture
Co-culture

Figure S6 to Figure 6. Neuron to glia GlcCer transport is evolutionarily conserved in human cells

(**A**) NBD-GlcCer labeling and internalization. (a-b) Neurons are incubated with NBD-GlcCer at 4°C for 30 minutes (Time 30), which labels the plasma membrane of neurons. (c-d) Labeled neurons are then incubated in ACSF without NBD-GlcCer at 37°C for 30 min (Time 60). The NBD-GlcCer is mostly internalized into the cytosol of labeled neurons. (e-g) The levels of cytosolic NBD-GlcCer remain unchanged for 30 min (Time 90). (**B**) Quantification of relative NBD-GlcCer intensity shown in Figure 6C, a-j. (**C**) Western blots to document that GBA1 is reduced in non-labeled glia by two individual siRNAs. (**D**) Quantification of relative NBD-GlcCer intensity shown in Figure 6C, a-b, k-r.

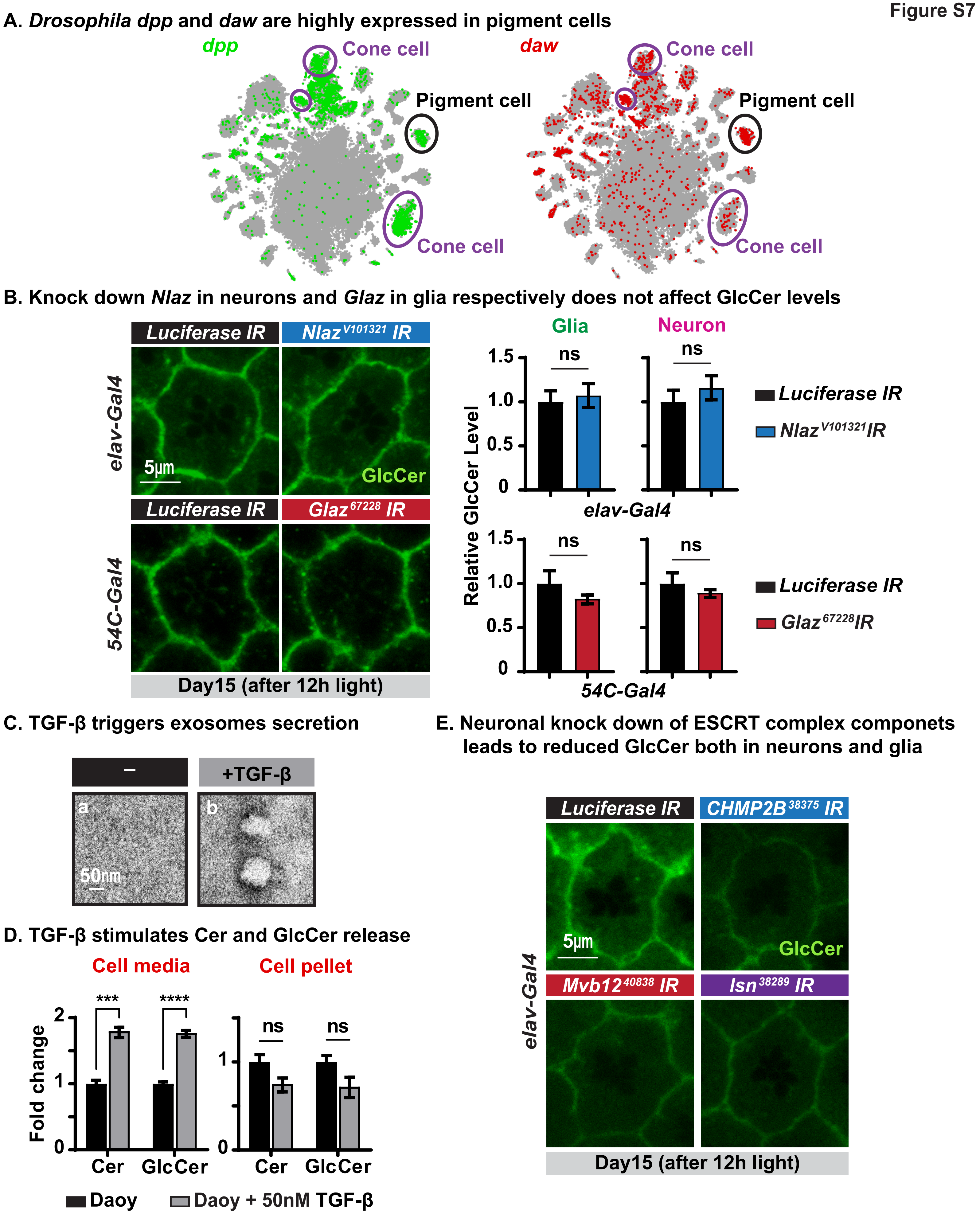
A. Drosophila dpp and daw are highly expressed in pigment cells
dpp
daw
Cone cell
Pigment cell
Cone cell
B. Knock down Nlaz in neurons and Glaz in glia respectively does not affect GlcCer levels
Luciferase IR
Nlaz V101321 IR
Glaz 67228 IR
elav-Gal4
54C-Gal4
5μm
GlcCer
Day15 (after 12h light)
Glia
Neuron
ns
Relative GlcCer Level
1.5
1.0
0.5
0
C. TGF-β triggers exosomes secretion
–
+TGF-β
a
b
50nm
D. TGF-β stimulates Cer and GlcCer release
Cell media
Cell pellet
***
****
ns
Fold change
2
1
0
Cer
GlcCer
Daoy
Daoy + 50nM TGF-β
E. Neuronal knock down of ESCRT complex componets leads to reduced GlcCer both in neurons and glia
Luciferase IR
CHMP2B 38375 IR
Mvb12 40838 IR
Isn 38289 IR
elav-Gal4
5μm
GlcCer
Day15 (after 12h light)

Figure S7 to Figure 7. The transport of GlcCer enriched exosomes from neurons to glia may not rely on lipoprotein particles

(**A**) Drosophila *daw* and *dpp* are highly expressed in cone and pigment cells. Data from large-scale scRNA-seq SCope database (10X head dataset, stringent version) suggest that *dpp*, a member in a TGF-β/BMP family in the *Drosophila*, is highly expressed in cone and pigment cells. Cone cells and pigments cells are two types of glia in the fly visual system (*39*, *40*, *87*). (**B**) Neuronal knock down of *Nlaz* and glial knock down of *Glaz*, respectively, do not affect the levels of GlcCer both in neurons and glia. Relative GlcCer levels are quantified on right. Error bars represent SEM ($n \geq 9$). (**C**) TGF-β triggers exosomes secretion. TEM images of purified exosomes from media of cells that were treated with and without 50nM TGF-β for one hour. Exosomes purified from media of cells that were treated with TGF-β have a diameter around 80 nm (b). However, exosomes are not detected form media of cells that were not treated with TGF-β (a). (N =3) (**D**) TGF-β stimulates Ceramide (Cer) and GlcCer release. Lipidomics are performed on cell media and cell pellets that are treated with and without 50nM TGF-β for one hour. Significant increased levels of Cer and GlcCer are observed in the cell culture media treated with TGF-β than the non-treated cell culture media, indicating that TGF-β promotes Cer and GlcCer secretion. However, The levels of Cer and GlcCer are not changed in cell pellets, suggesting that TGF-β does not affect the endogenous cellular Cer and GlcCer metabolism. Fold changes of Cer and GlcCer level is shown. Error bars represent SEM ($n = 3$); ***$p < 0.001$, ****$p < 0.0001$. (**E**) Neuronal knock down of ESCRT complex components *CHMP2B*, *Mvb12*, and *lsn* in fly photoreceptor neurons causes reduced levels of GlcCer both in neurons and glia.

REFERENCES AND NOTES

1. B. Chaurasia, S. A. Summers, Ceramides in metabolism: Key lipotoxic players. *Annu. Rev. Physiol.* **83**, 1–28 (2020).

2. M. L. Kraft, Sphingolipid organization in the plasma membrane and the mechanisms that influence it. *Front. Cell Dev. Biology.* **4**, 154 (2017).

3. D. K. Breslow, J. S. Weissman, Membranes in balance: Mechanisms of sphingolipid homeostasis. *Mol. Cell* **40**, 267–279 (2010).

4. C. R. Gault, L. M. Obeid, Y. A. Hannun, An overview of sphingolipid metabolism: From synthesis to breakdown. *Adv. Exp. Med. Biol.* **688**, 1–23 (2010).

5. N. Kudo, K. Kumagai, N. Tomishige, T. Yamaji, S. Wakatsuki, M. Nishijima, K. Hanada, R. Kato, Structural basis for specific lipid recognition by CERT responsible for nonvesicular trafficking of ceramide. *Proc. Natl. Acad. Sci. U.S.A.* **105**, 488–493 (2008).

6. R. P. Rao, C. Yuan, J. C. Allegood, S. S. Rawat, M. B. Edwards, X. Wang, A. H. Merrill, U. Acharya, J. K. Acharya, Ceramide transfer protein function is essential for normal oxidative stress response and lifespan. *Proc. Natl. Acad. Sci. U.S.A.* **104**, 11364–11369 (2007).

7. F. M. Goñi, A. Alonso, Effects of ceramide and other simple sphingolipids on membrane lateral structure. *Biochim. Biophys. Acta B Biomembr.* **1788**, 169–177 (2009).

8. T.-Y. Wang, J. R. Silvius, Sphingolipid partitioning into ordered domains in cholesterol-free and cholesterol-containing lipid bilayers. *Biophys. J.* **84**, 367–378 (2003).

9. S. Chiantia, N. Kahya, J. Ries, P. Schwille, Effects of ceramide on liquid-ordered domains investigated by simultaneous AFM and FCS. *Biophys. J.* **90**, 4500–4508 (2006).

10. M. Vos, M. Dulovic-Mahlow, F. Mandik, L. Frese, Y. Kanana, S. H. Diaw, J. Depperschmidt, C. Böhm, J. Rohr, T. Lohnau, I. R. König, C. Klein, Ceramide accumulation induces mitophagy and impairs β-oxidation in PINK1 deficiency. *Proc. Natl. Acad. Sci. U.S.A.* **118**, e2025347118 (2021).

11. G. Lin, P.-T. Lee, K. Chen, D. Mao, K. L. Tan, Z. Zuo, W.-W. Lin, L. Wang, H. J. Bellen, Phospholipase PLA2G6, a parkinsonism-associated gene, affects Vps26 and Vps35, retromer function, and ceramide levels, similar to α-synuclein gain. *Cell Metab.* **28**, 605–618.e6 (2018).

12. J. Rohrbough, E. Rushton, L. Palanker, E. Woodruff, H. J. G. Matthies, U. Acharya, J. K. Acharya, K. Broadie, Ceramidase regulates synaptic vesicle exocytosis and trafficking. *J. Neurosci.* **24**, 7789–7803 (2004).

13. M. L. Allende, R. L. Proia, Simplifying complexity: Genetically resculpting glycosphingolipid synthesis pathways in mice to reveal function. *Glycoconj. J.* **31**, 613–622 (2014).

14. L. A. Robak, I. E. Jansen, J. van Rooij, A. G. Uitterlinden, R. Kraaij, J. Jankovic, P. Heutink, J. M. Shulman; International Parkinson's Disease Genomics Consortium (IPDGC), M. A. Nalls,

V. Plagnol, D. G. Hernandez, M. Sharma, U.-M. Sheerin, M. Saad, J. S.-Sánchez, C. Schulte, S. Lesage, S. Sveinbjörnsdóttir, S. Arepalli, R. Barker, Y. Ben, H. W. Berendse, D. Berg, K. Bhatia, R. M. A. de Bie, A. Biffi, B. Bloem, Z. Bochdanovits, M. Bonin, J. M. Bras, K. Brockmann, J. Brooks, D. J. Burn, E. Majounie, G. Charlesworth, C. Lungu, H. Chen, P. F. Chinnery, S. Chong, C. E. Clarke, M. R. Cookson, J. M. Cooper, J. C. Corvol, C. Counsell, P. Damier, J.-F. Dartigues, P. Deloukas, G. Deuschl, D. T. Dexter, K. D. van Dijk, A. Dillman, F. Durif, A. Dürr, S. Edkins, J. R. Evans, T. Foltynie, J. Dong, M. Gardner, J. R. Gibbs, A. Goate, E. Gray, R. Guerreiro, C. Harris, J. J. van Hilten, A. Hofman, A. Hollenbeck, J. Holton, M. Hu, X. Huang, I. Wurster, W. Mätzler, G. Hudson, S. E. Hunt, J. Huttenlocher, T. Illig, P. V. Jónsson, J.-C. Lambert, C. Langford, A. Lees, P. Lichtner, P. Limousin, G. Lopez, D. Lorenz, A. McNeill, C. Moorby, M. Moore, H. R. Morris, K. E. Morrison, V. Escott-Price, E. Mudanohwo, S. S. O'Sullivan, J. Pearson, J. S. Perlmutter, H. Pétursson, P. Pollak, B. Post, S. Potter, B. Ravina, T. Revesz, O. Riess, F. Rivadeneira, P. Rizzu, M. Ryten, S. Sawcer, A. Schapira, H. Scheffer, K. Shaw, I. Shoulson, J. Shulman, E. Sidransky, C. Smith, C. C. A. Spencer, H. Stefánsson, F. Bettella, J. D. Stockton, A. Strange, K. Talbot, C. M. Tanner, A. Tashakkori-Ghanbaria, F. Tison, D. Trabzuni, B. J. Traynor, A. G. Uitterlinden, D. Velseboer, M. Vidailhet, R. Walker, B. van de Warrenburg, M. Wickremaratchi, N. Williams, C. H. Williams-Gray, S. Winder-Rhodes, K. Stefánsson, M. Martinez, N. W. Wood, J. Hardy, P. Heutink, A. Brice, T. Gasser, A. B. Singleton, Excessive burden of lysosomal storage disorder gene variants in Parkinson's disease. *Brain* **140**, 3191–3203 (2017).

15. G. Lin, L. Wang, P. C. Marcogliese, H. J. Bellen, Sphingolipids in the pathogenesis of Parkinson's disease and parkinsonism. *Trends Endocrinol. Metab.* **30**, 106–117 (2018).

16. E. Sidransky, Gaucher disease: Complexity in a "simple" disorder. *Mol. Genet. Metab.* **83**, 6–15 (2004).

17. K. Wong, E. Sidransky, A. Verma, T. Mixon, G. D. Sandberg, L. K. Wakefield, A. Morrison, A. Lwin, C. Colegial, J. M. Allman, R. Schiffmann, Neuropathology provides clues to the pathophysiology of Gaucher disease. *Mol. Genet. Metab.* **82**, 192–207 (2004).

18. D. Chang, M. A. Nalls, I. B. Hallgrímsdóttir, J. Hunkapiller, M. van der Brug, F. Cai; International Parkinson's Disease Genomics Consortium, 23andMe Research Team, G. A. Kerchner, G. Ayalon, B. Bingol, M. Sheng, D. Hinds, T. W. Behrens, A. B. Singleton, T. R. Bhangale, R. R. Graham, A meta-analysis of genome-wide association studies identifies 17 new Parkinson's disease risk loci. *Nat. Genet.* **49**, 1511–1516 (2017).

19. E. Sidransky, M. A. Nalls, J. O. Aasly, J. Aharon-Peretz, G. Annesi, E. R. Barbosa, A. Bar-Shira, D. Berg, J. Bras, A. Brice, C.-M. Chen, L. N. Clark, C. Condroyer, E. V. D. Marco, A. Dürr, M. J. Eblan, S. Fahn, M. J. Farrer, H.-C. Fung, Z. Gan-Or, T. Gasser, R. Gershoni-Baruch, N. Giladi, A. Griffith, T. Gurevich, C. Januario, P. Kropp, A. E. Lang, G.-J. Lee-Chen, S. Lesage, K. Marder, I. F. Mata, A. Mirelman, J. Mitsui, I. Mizuta, G. Nicoletti, C. Oliveira, R. Ottman, A. Orr-Urtreger, L. V. Pereira, A. Quattrone, E. Rogaeva, A. Rolfs, H. Rosenbaum, R. Rozenberg, A. Samii, T. Samaddar, C. Schulte, M. Sharma, A. Singleton, M. Spitz, E.-K. Tan, N. Tayebi, T. Toda, A. R. Troiano, S. Tsuji, M. Wittstock, T. G. Wolfsberg, Y.-R. Wu, C. P.

Zabetian, Y. Zhao, S. G. Ziegler, Multicenter analysis of glucocerebrosidase mutations in Parkinson's disease. *N. Engl. J. Med.* **361**, 1651–1661 (2009).

20. J. Do, C. McKinney, P. Sharma, E. Sidransky, Glucocerebrosidase and its relevance to Parkinson disease. *Mol. Neurodegener.* **14**, 36 (2019).

21. M. Y. Davis, K. Trinh, R. E. Thomas, S. Yu, A. A. Germanos, B. N. Whitley, S. P. Sardi, T. J. Montine, L. J. Pallanck, Glucocerebrosidase deficiency in drosophila results in α-synuclein-independent protein aggregation and neurodegeneration. *PLOS Genet.* **12**, e1005944 (2016).

22. K. J. Kinghorn, S. Grönke, J. I. Castillo-Quan, N. S. Woodling, L. Li, E. Sirka, M. Gegg, K. Mills, J. Hardy, I. Bjedov, L. Partridge, A drosophila model of neuronopathic gaucher disease demonstrates lysosomal-autophagic defects and altered mTOR signalling and is functionally rescued by rapamycin. *J. Neurosci.* **36**, 11654–11670 (2016).

23. O. Cabasso, S. Paul, O. Dorot, G. Maor, O. Krivoruk, M. Pasmanik-Chor, M. Mirzaian, M. Ferraz, J. Aerts, M. Horowitz, *Drosophila melanogaster* mutated in its GBA1b ortholog recapitulates neuronopathic gaucher disease. *J. Clin. Med.* **8**, 1420 (2019).

24. M. Keatinge, H. Bui, A. Menke, Y.-C. Chen, A. M. Sokol, Q. Bai, F. Ellett, M. D. Costa, D. Burke, M. Gegg, L. Trollope, T. Payne, A. McTighe, H. Mortiboys, S. de Jager, H. Nuthall, M.-S. Kuo, A. Fleming, A. H. V. Schapira, S. A. Renshaw, J. R. Highley, A. Chacinska, P. Panula, E. A. Burton, M. J. O'Neill, O. Bandmann, Glucocerebrosidase 1 deficient *Danio rerio* mirror key pathological aspects of human Gaucher disease and provide evidence of early microglial activation preceding alpha-synuclein-independent neuronal cell death. *Hum. Mol. Genet.* **24**, 6640–6652 (2015).

25. G. B. Sinclair, G. Jevon, K. E. Colobong, D. R. Randall, F. Y. M. Choy, L. A. Clarke, Generation of a conditional knockout of murine glucocerebrosidase: Utility for the study of Gaucher disease. *Mol. Genet. Metab.* **90**, 148–156 (2007).

26. L. D. Osellame, A. A. Rahim, I. P. Hargreaves, M. E. Gegg, A. Richard-Londt, S. Brandner, S. N. Waddington, A. H. V. Schapira, M. R. Duchen, Mitochondria and quality control defects in a mouse model of gaucher disease—Links to Parkinson's disease. *Cell Metab.* **17**, 941–953 (2013).

27. I. B. Enquist, C. L. Bianco, A. Ooka, E. Nilsson, J.-E. Månsson, M. Ehinger, J. Richter, R. O. Brady, D. Kirik, S. Karlsson, Murine models of acute neuronopathic Gaucher disease. *Proc. Natl. Acad. Sci. U.S.A.* **104**, 17483–17488 (2007).

28. modENCODE Consortium, S. Roy, J. Ernst, P. V. Kharchenko, P. Kheradpour, N. Negre, M. L. Eaton, J. M. Landolin, C. A. Bristow, L. Ma, M. F. Lin, S. Washietl, B. I. Arshinoff, F. Ay, P. E. Meyer, N. Robine, N. L. Washington, L. D. Stefano, E. Berezikov, C. D. Brown, R. Candeias, J. W. Carlson, A. Carr, I. Jungreis, D. Marbach, R. Sealfon, M. Y. Tolstorukov, S. Will, A. A. Alekseyenko, C. Artieri, B. W. Booth, A. N. Brooks, Q. Dai, C. A. Davis, M. O. Duff, X. Feng, A. A. Gorchakov, T. Gu, J. G. Henikoff, P. Kapranov, R. Li, H. K. MacAlpine, J. Malone, A. Minoda, J. Nordman, K. Okamura, M. Perry, S. K. Powell, N. C. Riddle, A. Sakai, A.

Samsonova, J. E. Sandler, Y. B. Schwartz, N. Sher, R. Spokony, D. Sturgill, M. van Baren, K. H. Wan, L. Yang, C. Yu, E. Feingold, P. Good, M. Guyer, R. Lowdon, K. Ahmad, J. Andrews, B. Berger, S. E. Brenner, M. R. Brent, L. Cherbas, S. C. R. Elgin, T. R. Gingeras, R. Grossman, R. A. Hoskins, T. C. Kaufman, W. Kent, M. I. Kuroda, T. Orr-Weaver, N. Perrimon, V. Pirrotta, J. W. Posakony, B. Ren, S. Russell, P. Cherbas, B. R. Graveley, S. Lewis, G. Micklem, B. Oliver, P. J. Park, S. E. Celniker, S. Henikoff, G. H. Karpen, E. C. Lai, D. M. MacAlpine, L. D. Stein, K. P. White, M. Kellis, D. Acevedo, R. Auburn, G. Barber, H. J. Bellen, E. P. Bishop, T. D. Bryson, A. Chateigner, J. Chen, H. Clawson, C. L. G. Comstock, S. Contrino, L. C. DeNapoli, Q. Ding, A. Dobin, M. H. Domanus, J. Drenkow, S. Dudoit, J. Dumais, T. Eng, D. Fagegaltier, S. E. Gadel, S. Ghosh, F. Guillier, D. Hanley, G. J. Hannon, K. D. Hansen, E. Heinz, A. S. Hinrichs, M. Hirst, S. Jha, L. Jiang, Y. L. Jung, H. Kashevsky, C. D. Kennedy, E. T. Kephart, L. Langton, O.-K. Lee, S. Li, Z. Li, W. Lin, D. Linder-Basso, P. Lloyd, R. Lyne, S. E. Marchetti, M. Marra, N. R. Mattiuzzo, S. McKay, F. Meyer, D. Miller, S. W. Miller, R. A. Moore, C. A. Morrison, J. A. Prinz, M. Rooks, R. Moore, K. M. Rutherford, P. Ruzanov, D. A. Scheftner, L. Senderowicz, P. K. Shah, G. Shanower, R. Smith, E. O. Stinson, S. Suchy, A. E. Tenney, F. Tian, K. J. T. Venken, H. Wang, R. White, J. Wilkening, A. T. Willingham, C. Zaleski, Z. Zha, D. Zhang, Y. Zhao, J. Zieba, Identification of functional elements and regulatory circuits by drosophila modENCODE. *Science* **330**, 1787–1797 (2010).

29. V. R. Chintapalli, J. Wang, J. A. T. Dow, Using FlyAtlas to identify better *Drosophila melanogaster* models of human disease. *Nat. Genet.* **39**, 715–720 (2007).

30. J. Borycz, J. A. Borycz, A. Kubów, V. Lloyd, I. A. Meinertzhagen, Drosophila ABC transporter mutants white, brown and scarlet have altered contents and distribution of biogenic amines in the brain. *J. Exp. Biol.* **211**, 3454–3466 (2008).

31. S. M. Mackenzie, A. J. Howells, G. B. Cox, G. D. Ewart, Sub-cellular localisation of the white/scarlet ABC transporter to pigment granule membranes within the compound eye of *Drosophila melanogaster*. *Genetica* **108**, 239–252 (2000).

32. P.-T. Lee, J. Zirin, O. Kanca, W.-W. Lin, K. L. Schulze, D. Li-Kroeger, R. Tao, C. Devereaux, Y. Hu, V. Chung, Y. Fang, Y. He, H. Pan, M. Ge, Z. Zuo, B. E. Housden, S. E. Mohr, S. Yamamoto, R. W. Levis, A. C. Spradling, N. Perrimon, H. J. Bellen, A gene-specific T2A-GAL4 library for Drosophila. *eLife* **7**, e35574 (2018).

33. F. Diao, B. H. White, A novel approach for directing transgene expression in drosophila: T2A-Gal4 in-frame fusion. *Genetics* **190**, 1139–1144 (2012).

34. E. Ryder, M. Ashburner, R. Bautista-Llacer, J. Drummond, J. Webster, G. Johnson, T. Morley, Y. S. Chan, F. Blows, D. Coulson, G. Reuter, H. Baisch, C. Apelt, A. Kauk, T. Rudolph, M. Kube, M. Klimm, C. Nickel, J. Szidonya, P. Maróy, M. Pal, Å. Rasmuson-Lestander, K. Ekström, H. Stocker, C. Hugentobler, E. Hafen, D. Gubb, G. Pflugfelder, C. Dorner, B. Mechler, H. Schenkel, J. Marhold, F. Serras, M. Corominas, A. Punset, J. Roote, S. Russell, The DrosDel deletion collection: A Drosophila genomewide chromosomal deficiency resource. *Genetics* **177**, 615–629 (2007).

35. K. J. T. Venken, J. W. Carlson, K. L. Schulze, H. Pan, Y. He, R. Spokony, K. H. Wan, M. Koriabine, P. J. de Jong, K. P. White, H. J. Bellen, R. A. Hoskins, Versatile P(acman) BAC libraries for transgenesis studies in *Drosophila melanogaster*. *Nat. Methods* **6**, 431–434 (2009).

36. K. Yao, K. White, Neural specificity of elav expression: Defining a Drosophila promoter for directing expression to the nervous system. *J. Neurochem.* **63**, 41–51 (1994).

37. T. Stork, R. Bernardos, M. R. Freeman, Analysis of glial cell development and function in Drosophila. *Cold Spring Harb. Protoc.* **2012**, 1–17 (2012).

38. C. J. Evans, J. M. Olson, K. T. Ngo, E. Kim, N. E. Lee, E. Kuoy, A. N. Patananan, D. Sitz, P. Tran, M.-T. Do, K. Yackle, A. Cespedes, V. Hartenstein, G. B. Call, U. Banerjee, G-TRACE: Rapid Gal4-based cell lineage analysis in Drosophila. *Nat. Methods* **6**, 603–605 (2009).

39. L. Liu, K. Zhang, H. Sandoval, S. Yamamoto, M. Jaiswal, E. Sanz, Z. Li, J. Hui, B. H. Graham, A. Quintana, H. J. Bellen, Glial lipid droplets and ROS induced by mitochondrial defects promote neurodegeneration. *Cell* **160**, 177–190 (2015).

40. M. A. Charlton-Perkins, E. D. Sendler, E. K. Buschbeck, T. A. Cook, Multifunctional glial support by Semper cells in the Drosophila retina. *PLOS Genet.* **13**, e1006782 (2017).

41. A. H. Brand, N. Perrimon, Targeted gene expression as a means of altering cell fates and generating dominant phenotypes. *Development* **118**, 401–415 (1993).

42. R. Nagaraj, U. Banerjee, Combinatorial signaling in the specification of primary pigment cells in the Drosophila eye. *Development* **134**, 825–831 (2007).

43. P. Dolph, A. Nair, P. Raghu, Electroretinogram recordings of Drosophila. *Cold Spring Harb. Protoc.* **2011**, pdb.prot5550 (2011).

44. R. N. Alcalay, O. A. Levy, C. H. Waters, S. Fahn, B. Ford, S.-H. Kuo, P. Mazzoni, M. W. Pauciulo, W. C. Nichols, Z. Gan-Or, G. A. Rouleau, W. K. Chung, P. Wolf, P. Oliva, J. Keutzer, K. Marder, X. Zhang, Glucocerebrosidase activity in Parkinson's disease with and without GBA mutations. *Brain* **138**, 2648–2658 (2015).

45. S. Roessingh, R. Stanewsky, The Drosophila TRPA1 channel and neuronal circuits controlling rhythmic behaviours and sleep in response to environmental temperature. *Int. J. Mol. Sci.* **18**, 2028 (2017).

46. X. Chen, R. Rahman, F. Guo, M. Rosbash, Genome-wide identification of neuronal activity-regulated genes in Drosophila. *eLife* **5**, e19942 (2016).

47. M. Verderame, D. Alcorta, M. Egnor, K. Smith, R. Pollack, Cytoskeletal F-actin patterns quantitated with fluorescein isothiocyanate-phalloidin in normal and transformed cells. *Proc. Natl. Acad. Sci. U.S.A.* **77**, 6624–6628 (1980).

48. D. Spira, J. Stypmann, D. J. Tobin, I. Petermann, C. Mayer, S. Hagemann, O. Vasiljeva, T. Günther, R. Schüle, C. Peters, T. Reinheckel, Cell type-specific functions of the lysosomal protease cathepsin L in the heart*. *J. Biol. Chem.* **282**, 37045–37052 (2007).

49. H. Li, J. Janssens, M. D. Waegeneer, S. S. Kolluru, K. Davie, V. Gardeux, W. Saelens, F. P. A. David, M. Brbić, K. Spanier, J. Leskovec, C. N. McLaughlin, Q. Xie, R. C. Jones, K. Brueckner, J. Shim, S. G. Tattikota, F. Schnorrer, K. Rust, T. G. Nystul, Z. Carvalho-Santos, C. Ribeiro, S. Pal, S. Mahadevaraju, T. M. Przytycka, A. M. Allen, S. F. Goodwin, C. W. Berry, M. T. Fuller, H. White-Cooper, E. L. Matunis, S. DiNardo, A. Galenza, L. E. O'Brien; J. A. T. Dow, F. C. A. Consortium§, H. Jasper, B. Oliver, N. Perrimon, B. Deplancke, S. R. Quake, L. Luo, S. Aerts, D. Agarwal, Y. Ahmed-Braimah, M. Arbeitman, M. M. Ariss, J. Augsburger, K. Ayush, C. C. Baker, T. Banisch, K. Birker, R. Bodmer, B. Bolival, S. E. Brantley, J. A. Brill, N. C. Brown, N. A. Buehner, X. T. Cai, R. Cardoso-Figueiredo, F. Casares, A. Chang, T. R. Clandinin, S. Crasta, C. Desplan, A. M. Detweiler, D. B. Dhakan, E. Donà, S. Engert, S. Floc'hlay, N. George, A. J. González-Segarra, A. K. Groves, S. Gumbin, Y. Guo, D. E. Harris, Y. Heifetz, S. L. Holtz, F. Horns, B. Hudry, R.-J. Hung, Y. N. Jan, J. S. Jaszczak, G. S. X. E. Jefferis, J. Karkanias, T. L. Karr, N. S. Katheder, J. Kezos, A. A. Kim, S. K. Kim, L. Kockel, N. Konstantinides, T. B. Kornberg, H. M. Krause, A. T. Labott, M. Laturney, R. Lehmann, S. Leinwand, J. Li, J. S. S. Li, K. Li, K. Li, L. Li, T. Li, M. Litovchenko, H.-H. Liu, Y. Liu, T.-C. Lu, J. Manning, A. Mase, M. Matera-Vatnick, N. R. Matias, C. E. McDonough-Goldstein, A. McGeever, A. D. McLachlan, P. Moreno-Roman, N. Neff, M. Neville, S. Ngo, T. Nielsen, C. E. O'Brien, D. Osumi-Sutherland, M. N. Özel, I. Papatheodorou, M. Petkovic, C. Pilgrim, A. O. Pisco, C. Reisenman, E. N. Sanders, G. dos Santos, K. Scott, A. Sherlekar, P. Shiu, D. Sims, R. V. Sit, M. Slaidina, H. E. Smith, G. Sterne, Y.-H. Su, D. Sutton, M. Tamayo, M. Tan, I. Tastekin, C. Treiber, D. Vacek, G. Vogler, S. Waddell, W. Wang, R. I. Wilson, M. F. Wolfner, Y.-C. E. Wong, A. Xie, J. Xu, S. Yamamoto, J. Yan, Z. Yao, K. Yoda, R. Zhu, R. P. Zinzen, Fly Cell Atlas: A single-nucleus transcriptomic atlas of the adult fruit fly. *Science* **375**, eabk2432 (2022).

50. P. T. Tarr, E. J. Tarling, D. D. Bojanic, P. A. Edwards, Á. Baldán, Emerging new paradigms for ABCG transporters. *Biochim. Biophys. Acta Mol. Cell Biol. Lipids* **1791**, 584–593 (2009).

51. M. J. Ferreiro, C. Pérez, M. Marchesano, S. Ruiz, A. Caputi, P. Aguilera, R. Barrio, R. Cantera, *Drosophila melanogaster* white mutant w1118 undergo retinal degeneration. *Front. Neurosci.* **11**, 732 (2018).

52. T. E. Lloyd, R. Atkinson, M. N. Wu, Y. Zhou, G. Pennetta, H. J. Bellen, Hrs regulates endosome membrane invagination and tyrosine kinase receptor signaling in Drosophila. *Cell* **108**, 261–269 (2002).

53. H. D. E. Booth, W. D. Hirst, R. Wade-Martins, The role of astrocyte dysfunction in Parkinson's disease pathogenesis. *Trends Neurosci.* **40**, 358–370 (2017).

54. Y. Zhang, K. Chen, S. A. Sloan, M. L. Bennett, A. R. Scholze, S. O'Keeffe, H. P. Phatnani, P. Guarnieri, C. Caneda, N. Ruderisch, S. Deng, S. A. Liddelow, C. Zhang, R. Daneman, T. Maniatis, B. A. Barres, J. Q. Wu, An RNA-sequencing transcriptome and splicing database of glia, neurons, and vascular cells of the cerebral cortex. *J. Neurosci.* **34**, 11929–11947 (2014).

55. A. Casciati, M. Tanori, R. Manczak, S. Saada, B. Tanno, P. Giardullo, E. Porcù, E. Rampazzo, L. Persano, G. Viola, C. Dalmay, F. Lalloué, A. Pothier, C. Merla, M. Mancuso,

Human medulloblastoma cell lines: Investigating on cancer stem cell-like phenotype. *Cancer* **12**, 226 (2020).

56. F. Boscia, C. D'Avanzo, A. Pannaccione, A. Secondo, A. Casamassa, L. Formisano, N. Guida, S. Sokolow, A. Herchuelz, L. Annunziato, Silencing or knocking out the Na^+/$Ca2^+$ exchanger-3 (NCX3) impairs oligodendrocyte differentiation. *Cell Death Differ.* **19**, 562–572 (2012).

57. X. Zheng, H. Baker, W. S. Hancock, F. Fawaz, M. McCaman, E. Pungor, Proteomic analysis for the assessment of different lots of fetal bovine serum as a raw material for cell culture. Part IV. Application of proteomics to the manufacture of biological drugs. *Biotechnol. Prog.* **22**, 1294–1300 (2006).

58. J. Luo, P. P. Ho, M. S. Buckwalter, T. Hsu, L. Y. Lee, H. Zhang, D.-K. Kim, S.-J. Kim, S. S. Gambhir, L. Steinman, T. Wyss-Coray, Glia-dependent TGF-β signaling, acting independently of the TH17 pathway, is critical for initiation of murine autoimmune encephalomyelitis. *J. Clin. Invest.* **117**, 3306–3315 (2007).

59. D. A. Cox, Transforming growth factor-beta 3. *Cell Biol. Int.* **19**, 357–371 (1995).

60. B. Haley, D. Hendrix, V. Trang, M. Levine, A simplified miRNA-based gene silencing method for *Drosophila melanogaster*. *Dev. Biol.* **321**, 482–490 (2008).

61. L. Liu, K. R. MacKenzie, N. Putluri, M. Maletić-Savatić, H. J. Bellen, The glia-neuron lactate shuttle and elevated ROS promote lipid synthesis in neurons and lipid droplet accumulation in glia via APOE/D. *Cell Metab.* **26**, 719–737.e6 (2017).

62. R. E. Thomas, E. S. Vincow, G. E. Merrihew, M. J. MacCoss, M. Y. Davis, L. J. Pallanck, Glucocerebrosidase deficiency promotes protein aggregation through dysregulation of extracellular vesicles. *PLOS Genet.* **14**, e1007694 (2018).

63. L. Corrigan, S. Redhai, A. Leiblich, S.-J. Fan, S. M. W. Perera, R. Patel, C. Gandy, S. M. Wainwright, J. F. Morris, F. Hamdy, D. C. I. Goberdhan, C. Wilson, BMP-regulated exosomes from Drosophila male reproductive glands reprogram female behavior. *J. Cell Biol.* **206**, 671–688 (2014).

64. M. Logozzi, A. D. Milito, L. Lugini, M. Borghi, L. Calabrò, M. Spada, M. Perdicchio, M. L. Marino, C. Federici, E. Iessi, D. Brambilla, G. Venturi, F. Lozupone, M. Santinami, V. Huber, M. Maio, L. Rivoltini, S. Fais, High levels of exosomes expressing CD63 and caveolin-1 in plasma of melanoma patients. *PLOS ONE* **4**, e5219 (2009).

65. O. Schmidt, Y. Weyer, V. Baumann, M. A. Widerin, S. Eising, M. Angelova, A. Schleiffer, L. Kremser, H. Lindner, M. Peter, F. Fröhlich, D. Teis, Endosome and Golgi-associated degradation (EGAD) of membrane proteins regulates sphingolipid metabolism. *EMBO J.* **38**, e101433 (2019).

66. O. Schmidt, Y. Weyer, S. Sprenger, M. A. Widerin, S. Eising, V. Baumann, M. Angelova, R. Loewith, C. J. Stefan, M. W. Hess, F. Fröhlich, D. Teis, TOR complex 2 (TORC2) signaling and

the ESCRT machinery cooperate in the protection of plasma membrane integrity in yeast. *J. Biol. Chem.* **295**, 12028–12044 (2020).

67. F. N. Soria, M. Engeln, M. Martinez-Vicente, C. Glangetas, M. J. López-González, S. Dovero, B. Dehay, E. Normand, M. Vila, A. Favereaux, F. Georges, C. L. Bianco, E. Bezard, P.-O. Fernagut, Glucocerebrosidase deficiency in dopaminergic neurons induces microglial activation without neurodegeneration. *Hum. Mol. Genet.* **26**, 2603–2615 (2017).

68. M. X. Henderson, S. Sedor, I. McGeary, E. J. Cornblath, C. Peng, D. M. Riddle, H. L. Li, B. Zhang, H. J. Brown, M. F. Olufemi, D. S. Bassett, J. Q. Trojanowski, V. M. Y. Lee, Glucocerebrosidase activity modulates neuronal susceptibility to pathological α-synuclein insult. *Neuron* **105**, 822–836.e7 (2020).

69. I. L. Calandri, M. A. Hawkes, M. Marrodan, S. F. Ameriso, J. Correale, R. F. Allegri, The impact of an early strict nationwide lockdown on the pattern of consultation for neurological diseases. *J. Neurol. Sci.* **418**, 117084 (2020).

70. D. H. M. Chao, W. W. Kallemeijn, A. R. A. Marques, M. Orre, R. Ottenhoff, C. van Roomen, E. Foppen, M. C. Renner, M. Moeton, M. van Eijk, R. G. Boot, W. Kamphuis, E. M. Hol, J. Aten, H. S. Overkleeft, A. Kalsbeek, J. M. F. G. Aerts, Visualization of active glucocerebrosidase in rodent brain with high spatial resolution following in situ labeling with fluorescent activity based probes. *PLOS ONE* **10**, e0138107 (2015).

71. T. Yamashita, R. Wada, T. Sasaki, C. Deng, U. Bierfreund, K. Sandhoff, R. L. Proia, A vital role for glycosphingolipid synthesis during development and differentiation. *Proc. Natl. Acad. Sci. U.S.A.* **96**, 9142–9147 (1999).

72. R. Jennemann, R. Sandhoff, S. Wang, E. Kiss, N. Gretz, C. Zuliani, A. Martin-Villalba, R. Jäger, H. Schorle, M. Kenzelmann, M. Bonrouhi, H. Wiegandt, H.-J. Gröne, Cell-specific deletion of glucosylceramide synthase in brain leads to severe neural defects after birth. *Proc. Natl. Acad. Sci. U.S.A.* **102**, 12459–12464 (2005).

73. P. J. Brennan, R. V. V. Tatituri, M. Brigl, E. Y. Kim, A. Tuli, J. P. Sanderson, S. D. Gadola, F.-F. Hsu, G. S. Besra, M. B. Brenner, Invariant natural killer T cells recognize lipid self antigen induced by microbial danger signals. *Nat. Immunol.* **12**, 1202–1211 (2011).

74. Y.-Y. Liu, G. A. Patwardhan, P. Xie, X. Gu, A. E. Giuliano, M. C. Cabot, Glucosylceramide synthase, a factor in modulating drug resistance, is overexpressed in metastatic breast carcinoma. *Int. J. Oncol.* **39**, 425–431 (2011).

75. L. Saadat, J. L. Dupree, J. Kilkus, X. Han, M. Traka, R. L. Proia, G. Dawson, B. Popko, Absence of oligodendroglial glucosylceramide synthesis does not result in CNS myelin abnormalities or alter the dysmyelinating phenotype of CGT-deficient mice. *Glia* **58**, 391–398 (2010).

76. N. S. Radin, Glucosylceramide in the nervous system—A mini-review. *Neurochem. Res.* **19**, 533–540 (1994).

77. C. R. Mikulka, J. T. Dearborn, B. A. Benitez, A. Strickland, L. Liu, J. Milbrandt, M. S. Sands, Cell-autonomous expression of the acid hydrolase galactocerebrosidase. *Proc. Natl. Acad. Sci. U.S.A.* **117**, 9032–9041 (2020).

78. K. A. Jewett, R. E. Thomas, C. Q. Phan, B. Lin, G. Milstein, S. Yu, L. F. Bettcher, F. C. Neto, D. Djukovic, D. Raftery, L. J. Pallanck, M. Y. Davis, Glucocerebrosidase reduces the spread of protein aggregation in a *Drosophila melanogaster* model of neurodegeneration by regulating proteins trafficked by extracellular vesicles. *PLOS Genet.* **17**, e1008859 (2021).

79. M. E. Gegg, A. H. V. Schapira, The role of glucocerebrosidase in Parkinson disease pathogenesis. *FEBS J.* **285**, 3591–3603 (2018).

80. E.-J. Bae, N.-Y. Yang, M. Song, C. S. Lee, J. S. Lee, B. C. Jung, H.-J. Lee, S. Kim, E. Masliah, S. P. Sardi, S.-J. Lee, Glucocerebrosidase depletion enhances cell-to-cell transmission of α-synuclein. *Nat. Commun.* **5**, 4755 (2014).

81. J. F. Reyes, N. L. Rey, L. Bousset, R. Melki, P. Brundin, E. Angot, Alpha-synuclein transfers from neurons to oligodendrocytes. *Glia* **62**, 387–398 (2014).

82. A. K. Chouhan, C. Guo, Y.-C. Hsieh, H. Ye, M. Senturk, Z. Zuo, Y. Li, S. Chatterjee, J. Botas, G. R. Jackson, H. J. Bellen, J. M. Shulman, Uncoupling neuronal death and dysfunction in Drosophila models of neurodegenerative disease. *Acta Neuropathol. Commun.* **4**, 62 (2016).

83. G. M. Halliday, C. H. Stevens, Glia: Initiators and progressors of pathology in Parkinson's disease. *Mov. Disord.* **26**, 6–17 (2011).

84. S. M. Mackenzie, M. R. Brooker, T. R. Gill, G. B. Cox, A. J. Howells, G. D. Ewart, Mutations in the white gene of *Drosophila melanogaster* affecting ABC transporters that determine eye colouration. *Biochim. Biophys. Acta B Biomembr.* **1419**, 173–185 (1999).

85. S. Wang, K. L. Tan, M. A. Agosto, B. Xiong, S. Yamamoto, H. Sandoval, M. Jaiswal, V. Bayat, K. Zhang, W.-L. Charng, G. David, L. Duraine, K. Venkatachalam, T. G. Wensel, H. J. Bellen, The retromer complex is required for rhodopsin recycling and its loss leads to photoreceptor degeneration. *PLoS Biol.* **12**, e1001847 (2014).

86. Z. H. Alshehry, C. K. Barlow, J. M. Weir, Y. Zhou, M. J. McConville, P. J. Meikle, An efficient single phase method for the extraction of plasma lipids. *Metabolites* **5**, 389–403 (2015).

87. O. Strauss, The retinal pigment epithelium in visual function. *Physiol. Rev.* **85**, 845–881 (2005).
